# Erythrocytes as bioreactors to decrease excess ammonium concentration in blood

**DOI:** 10.1038/s41598-018-37828-5

**Published:** 2019-02-06

**Authors:** Eugeniy S. Protasov, Daria V. Borsakova, Yuliya G. Alexandrovich, Anatoliy V. Korotkov, Elena A. Kosenko, Andrey A. Butylin, Fazoil I. Ataullakhanov, Elena I. Sinauridze

**Affiliations:** 1 Dmitriy Rogachev National Medical Research Center of Pediatric Hematology, Oncology and Immunology, Ministry of Healthcare, Samory Mashela str., 1, GSP-7, Moscow, 117997 Russia; 2 Faculty of Physics, Moscow State University, Leninskie Gory, 1, build. 2, GSP-1, Moscow, 119991 Russia; 3 Center for Theoretical Problems of Physicochemical Pharmacology, Russian Academy of Sciences, Kosygina str., 4, Moscow, 119334 Russia; 4 Moscow Institute of Physics and Technology, Institutskiy per., 9, Dolgoprudny, Moscow region, 141701 Russia; 5 Institute of Theoretical and Experimental Biophysics, Russian Academy of Sciences, Institutskaya str., 3, Pushchino Moscow region, 142290 Russia

## Abstract

Increased blood ammonium concentrations cause neurological complications. Existing drugs are not always sufficiently effective. Alternatively, erythrocytes-bioreactors (EBRs) loaded with enzymes utilizing ammonium, were suggested for ammonium removal from blood. However all they worked only for a short period of time. The reasons for this were not investigated. In this study, EBR mathematical models were developed and analysed based on the reactions of glycolysis and different enzymes utilizing ammonium, which showed that the efficiency and duration of EBRs’ functioning could be limited due to low permeability of the cell membrane for some key substrates and products. A new enzyme system including glutamate dehydrogenase and alanine aminotransferase was proposed and realised experimentally, which was not limited by cell membrane permeability for glutamate and α-ketoglutarate due to creating metabolic pathway where these metabolites were produced and consumed cyclically. New bioreactors removed ammonium *in vitro* at the rate of 1.5 mmol/h × l_RBCs_ (for human bioreactors) and *in vivo* in a model of hyperammoniemia in mice at the rate of 2.0 mmol/h × l_RBCs_ (for mouse bioreactors), which correlated with model calculations. Experimental studies proved the proposed mathematical models are correct. Mathematical simulation of erythrocyte-bioreactors opens new opportunities for analysing the efficiency of any enzyme included in erythrocytes.

## Introduction

The aim of this study was to develop a new bioreactor based on human erythrocytes (EBR), to provide an effective ammonium removal from the blood of patients with hyperammonaemia. A number of acute and chronic liver diseases, as well as disorders associated with а deficiency of certain enzymes in the urea cycle, are accompanied by an increased blood ammonium concentration. Due to the toxicity of the ammonium for the central nervous system, patients with high blood ammonium levels develop hepatic encephalopathy, tremors, convulsions, and are at high risk for coma and death^1,2^. These states require an obligatory rapid correction. However, currently this correction using known existing medications is not always sufficiently effective (a decrease in ammonium concentrations to normal level (≤60 µM) in patients’ blood usually requires 2–10 days)^3–14^.

It is well known that erythrocytes may be carriers of some drugs^15–17^. They can also serve as bioreactors for the inclusion of enzyme preparations. Enzymes may function inside the cell provided that the necessary substrates are able to enter the erythrocyte from the plasma^18^. The encapsulation of the enzyme in erythrocytes was shown to protect it from immune system cells, thus reducing unwanted immune responses and increasing the half-life of the medication in the body compared to conventional intravenous administration^17,19–22^.

Bioreactors for ammonium removal based on mouse, sheep and human erythrocytes were investigated by various groups. They used only two enzymes: glutamine synthetase (GS) catalysing the formation of L-glutamine from L-glutamic acid and ammonium in the presence of ATP (reaction 1)^23,24^ or L-glutamate dehydrogenase (GDH) which catalyses the formation of L-glutamic acid from α-ketoglutarate and ammonium in the presence of NADPH (reaction (2), see below)^25–27^. Both enzymes were encapsulated in erythrocytes (RBCs) by the hypotonic dialysis-resealing method. These EBRs were shown to retain key metabolites after dialysis and to have a satisfactory survival. However, *in vivo* these EBRs effectively removed ammonium from the mice circulation only in the first 0.5–1 h^23,25^. After this time, the blood ammonium concentration decreased approximately at the same rate in both the experimental and the control animals, which received dialysed erythrocytes but without enzymes. This finding suggested that after 0.5–1 h, the enzymes inside the EBRs ceased to contribute to the process of ammonium consumption. The reasons for this, as well as the questions about the maximum possible rates of ammonium removal using EBRs were practically not analysed. To answer these challenges we developed the mathematical models of different EBRs, which allowed us to analyse the efficiency of previously proposed and other enzyme systems, which can use ammonium, and choose the most suitable system for creating ammocytes, i.e. EBRs to remove excess ammonium from the plasma. In the abovementioned studies, little attention was paid to the transport of the necessary reaction substrates and products through the RBC membrane. L-glutamic acid (GLU) almost does not pass through the membrane, and α-ketoglutarate (AKG) passes it quite slowly^28,29^. As a result, GLU and AKG may be almost completely depleted during reactions in EBRs loaded with GS or GDH, respectively. In contrast, GLU may accumulate in bioreactors with GDH. Changes in the participant concentrations should cause shifts of reaction equilibriums according to Le Châtelier’s principle. As a result, therefore, bioreactors might remove ammonium from the blood for a short period of time, until the reaction equilibrium completely shifts towards ammonium production. The operating time of bioreactors as cells removing ammonium depends on the equilibrium constants of each reaction, and on the closeness of the effective masses ratio of these reactions to corresponding equilibrium constants under physiological conditions.

The theoretical study helped us to answer questions about the stability and direction of the EBRs operation and to calculate the rates of ammonium removal *in vitro* in systems containing such EBRs. This approach can be used to analyse the effect of any enzyme included in erythrocytes.

For the model verification, the rate of ammonium decrease in the presence of the most promising (in accordance with our model) EBRs in the buffer system *in vitro* was investigated experimentally. These results were in good agreement with the theoretical predictions. We also showed that corresponding EBRs decreased ammonium level in mice with induced hyperammonemia.

## Methods

### Development of mathematical models of various bioreactors for ammonium removal

We developed mathematical models for human EBRs based on the following enzymes:

**EBR #1:** glutamine synthetase (GS, reaction (1)):

1 $${\rm{GLU}}+{{\rm{NH}}}_{4}^{+}+{\rm{ATP}}\leftrightarrow {\rm{GLN}}+{\rm{ADP}}+{{\rm{PO}}}_{4}^{-3}$$

**EBR #2**: glutamate dehydrogenase (GDH, reaction (2)):

2 $${\rm{AKG}}+{{\rm{NH}}}_{4}^{+}+{{\rm{H}}}^{+}+{\rm{NADPH}}({\rm{or}}\,{\rm{NADH}})\leftrightarrow {\rm{GLU}}+{\rm{NADP}}({\rm{or}}\,{\rm{NAD}})+{{\rm{H}}}_{{\rm{2}}}{\rm{O}}$$

**EBR #3**: alanine dehydrogenase (ADH, reaction (3)):

3 $${\rm{PYR}}+{{\rm{NH}}}_{4}^{+}+{{\rm{H}}}^{+}+{\rm{NADH}}\leftrightarrow {\rm{ALA}}+{\rm{NAD}}+{{\rm{H}}}_{2}{\rm{O}}$$

**EBR #4**: EBR based on the joint action of GDH (see reaction (2)) and alanine aminotransferase (AAT, reaction (4)):

4 $${\rm{GLU}}+{\rm{PYR}}\leftrightarrow {\rm{AKG}}+{\rm{ALA}}$$

where GLU, L-glutamic acid; GLN, L-glutamine; ATP and ADP, adenosine-5′-tri- and adenosine-5′-diphosphate, respectively; NH_4_^+^, ammonium ion; PO_4_^−3^, inorganic phosphate anion; AKG, α-ketoglutarate; NAD, NADP and NADH, NADPH, oxidised and reduced forms, respectively, of nicotinamide adenine dinucleotide and nicotinamide adenine dinucleotide phosphate, respectively; PYR, pyruvate; and ALA, alanine.

All models were systems of ordinary differential equations. The glycolysis model of Martinov *et al*. was used as a basis^30^. For our purposes, the model was somewhat modified.

The following assumptions were made:

1. The rate of hexokinase reaction was considered independent of the glucose concentration, since the Michaelis constant (K_M_) of hexokinase for glucose is ten times lower than the physiological glucose concentrations^30,31^.
2. The erythrocyte membrane was considered impenetrable for all the metabolites, excepting lactate (LAC), PYR, AKG, ALA, GLN and ammonium^28,29,32–35^ (Table 1).

**Table 1:** Concentrations and RBC membrane permeability coefficients (in physiological conditions) for the metabolites of glycolysis and of built-in reactions^a^.

Metabolite	Range of the experimentally measured concentrations into the cell, mmol/l_RBCs_	Range of the experimentally measured concentrations in plasma, mmol/l	Coefficient of permeability calculated from the literature data (K_p_), h^−1^	Initial values of metabolite intracellular concentration used in the calculations, mmol/l_RBCs_
Pyruvate	0.047–0.950^53,54^	0.317–0.355^43^	51.8^32^	0.07
Alanine	0.275–0.435^28^	0.300–0.373^28^	1.95^34^	0.3
Glutamate	0.212–0.463^28,35^	0.024–0.050^35^	0^28^	0.45
Glutamine	0.197–0.922^28,35,55^	0.447–0.776^28,35,55^	0.65^35^	0.625
α-Ketoglytarate	0.005 (model)^28^	0.000–0.093^56^	0.029^28,29^	0.005
Lactate	1.00–2.14^43,44,53^	0.9–1.5^44^	7.8^32^	1.2
(NH_3_ + NH_4_^+^)^b^	0.010–0.050^46,57^	0.010–0.050^46,57^	700000^33^	—

^a^The value of AKG concentration in RBC was calculated using mathematical model^28^.

^b^The sum concentration (ammonia + ammonium) in normal blood is presented. The concentration of ammonia is about 1% of this total concentration, therefore in this study we mean under the concentration of ammonium the total concentration of ammonia and ammonium. The equilibrium ammonium concentrations within the cell and in plasma are achieved very quickly.
3. The rates of transport of LAC, PYR and other metabolites penetrating across the cell membrane, were considered proportional to the concentration gradients for each of them, since this provides a good approximation for the description of the rates calculated from the precise kinetic mechanisms^32^.
4. Ammonium transport was considered to be sufficiently fast, so that its concentrations in plasma and cells remained in equilibrium at any time^33^.
5. The model did not include equations describing the trans-membrane potential.
6. The pools of adenylates and nicotinamide adenine dinucleotides were considered to be constant, since under normal physiological conditions the size of these pools in the erythrocyte changed slowly in comparison with the times that interest us.
7. The rate of NADPH oxidation in oxidative metabolism was considered to be constant.
8. The pentose phosphate pathway (PPP) is represented only by the glucose-6-phosphate dehydrogenase reaction, since this reaction limits the rate in PPP^36^.
9. The volume of erythrocyte was considered unchanged.

The kinetics of the metabolites inside the erythrocyte was calculated. Numerical solutions of the equations system were obtained by the Runge-Kutta method of the 4^th^-5^th^ order in the MATLAB program.

Complete systems of equations and the expressions for the rates of included enzymatic reactions are given in Supplementary Information (Tables S1–S8). The values of the metabolites’ concentrations and kinetic constants used are presented in Tables 1 and 2.

**Table 2:** The concentrations of glycolysis metabolites in erythrocytes, measured in various studies, as well as the steady-state concentrations of these metabolites obtained in the model (for normal physiological conditions)^a^.

Metabolite	Range of the intracellular concentration measured experimentally, mmol/l_RBCs_	Steady-state intracellular concentration calculated in model, mmol/l_RBCs_	References
G6P	0.02–0.11	0.073	^53, 54, 58– 64^
F6P	0.006–0.016	0.024	^53, 54, 59– 63^
FDP	0.002–0.03	0.0075	^53, 54, 58– 62, 64^
DAP	0.0076–0.035	0.032	^53, 54, 61, 62, 65^
GAP	0.0048–0.02	0.014	^59, 61, 63, 65^
1,3-DPG	(0.62–4.3) × 10^−4^	7.2 × 10^−4^	^61, 66^
2,3-DPG	4.17–5.7	4.57	^53, 54, 59– 61, 63– 65^
3-PG	0.055–0.069	0.045	^53, 54, 59– 61, 63^
2-PG	0.0055–0.012	0.011	^53, 54, 59– 61, 63^
PEP	0.012–0.018	0.0098	^53, 54, 59– 61, 63^
NAD	0.032–0.092	0.048	^48, 67^
NADH	0.001–0.054	0.002	^48, 67^
PО_4_	0.5–0.9	1	^53, 68, 69^
ATP	1.07–1.8	1.47	^60– 62, 64^
ADP	0.085–0.3	0.23	^60– 62, 64^
AMP	0.01–0.05	0.04	^60– 62, 64^
NADP	0.0003–0.023	0.0003	^48, 70– 72^
NADPH	0.035–0.06	0.0498	^48, 70– 72^
Na^+^ ions	9.2–12.6	10	^73, 74^

^a^For abbreviations, see legend to Fig. 1.

### Transport of the penetrating metabolites across the erythrocyte membrane

Despite of existence of the different mechanisms for transport of penetrating metabolites across the cell membrane, we described this transport very simplistically, using linear approximation by the equation:

5 $${V}_{transpA}={K}_{p}^{A}\times ({[A]}_{int}-{[A]}_{ext}),$$

where *V*_*transp A*_ is the transport rate for metabolite A, [*A*]_*int*_ and [*A*]_ext_ are the concentrations of this metabolite in erythrocyte and in the external medium, respectively; and *K*_*p*_^*A*^ is a permeability coefficient of A across the erythrocyte membrane. The used in the calculations values of the initial intracellular concentrations and the permeability coefficients of the metabolites are given in Table 1. The intracellular activity of all the enzymes was calculated per 1 litre of erythrocytes (l_RBCs_). The permeability coefficients were estimated from literature data using a summary metabolite influx rate into RBCs (by all known transport mechanisms) and the known plasma concentration of the corresponding metabolite.

### Ethics Statement

This study was approved by the Ethical Committee of the Center for Theoretical Problems of Physicochemical Pharmacology (Permit Number: 21-04-2009). All participants provided written informed consent before blood collection. The blood of healthy donors was received at the station of blood transfusion and was used without authentication. The housing and operating conditions for animals strictly satisfied the requirements of the Guide for Care and Use of the Laboratory Animals^37^. All efforts were made to minimise suffering in animals. All methods were carried out in accordance with the approved guidelines.

### Materials

All reagents used: L-glutamate dehydrogenase from bovine liver (type 1); alanine aminotransferase from porcine heart; rabbit muscle L-lactate dehydrogenase; glucose; adenine; inosine; alanine; NADН; phosphate buffered saline (PBS) containing 10 mM sodium phosphate buffer, 138 mM NaCl and 2.7 mM KCl (pH 7.4); α-ketoglutarate; bovine serum albumin (BSA); pyridoxal 5′-phosphate; sodium pyruvate, and inorganic salts were purchased from Sigma-Aldrich (St. Louis, MO, USA).

A line of Swiss mice (males weighing 25–30 g) from the Pushchino vivarium was used in *in vivo* experiments.

### Isolation of erythrocytes

The blood was collected in standard vacuum tubes Vacuette (Greiner Bio-One GmbH, Frickenhausen, Germany) with 3.2% (0.109 M) sodium citrate (citrate/blood ratio was 1/9) and centrifuged (1000 g, 8 min). Erythrocytes were then washed three times in a four-fold volume of PBS using centrifugation (1000 g, 8 min).

### Encapsulation of enzymes into erythrocytes

For the encapsulation of enzymes into RBCs a reversible hypoosmotic dialysis method was used (the method of hypoosmotic dialysis – resealing). Haematocrit (Ht) of the suspension of the original washed RBCs prepared as described above was adjusted to 70% by adding PBS. To 625 μl of distilled H_2_0, 20 μl of the original GDH suspension and 10 μl of the original suspension of AAT were added (the osmolality of the resulting solution of this enzyme mixture was ~300 mOsm/kg). The resulting mixture of enzymes (655 μl) was added to 1 ml of the suspension of the original RBCs (with Ht 70%). The final Ht of the resulting suspension was ~42%. The enzyme activity in this suspension was about 10 IU/ml_suspension_ and 5 IU/ml_suspension_ for GDH and AAT, respectively. This suspension was dialysed in bags (Cellusep T3, Membrane Filtration Products, Inc., Seguin, TX, USA) for 1 h at 4 °C against a hypoosmotic solution. The volume ratio of the sample to the hypoosmotic buffer was 1/100. The hypoosmotic solution contained 2 mM sodium phosphate buffer (pH 7.4), 0.5 mM KCl, 27 mM NaCl and had a total osmolality of 60–65 mOsm/kg. Osmolality of solutions and suspensions was measured on a Vapor Pressure Osmometer (Vapro 5600) (Wescor Inc., Logan, UT, USA).

After dialysis, the suspension was placed in a tube, and NADH and pyridoxal 5′-phosphate (cofactor of AAT) were added in final concentrations of 0.3 mM and 0.2 mM, respectively. The mixture was gently stirred and a hyperosmotic buffer (1020 mOsm/kg) was added in a volume of 1/2 volume of the dialysed suspension. This buffer contained 30 mM Na_2_HPO_4_, 30 mM inosine, 1.5 mM adenine, 8.4 mM glucose, 1.5 mM sodium pyruvate, 3 mM MgCl_2_, 55 mM NaCl, and 458 mM KCl (pH 7.4). The suspension was incubated at 37 °C for 30 min. The obtained ammocytes were washed in PBS solution five times by centrifugation for 8 min at 1000 g.

Ht and activity (for each of the enzymes) were measured in the initial suspension (with enzymes, but before dialysis) and in the final suspension of the resulting EBRs.

Three parameters were used to characterise the efficiency of inclusion:

1. Cell yield (C), which is the percentage of cells retained in the suspension after the procedure, from their initial amount in the system:
  6 $$C( \% )={{\rm{V}}}_{{\rm{susp}}{\rm{EBRs}}}\times {{\rm{Ht}}}_{{\rm{susp}}{\rm{EBRs}}}\times {\mathrm{100}/({\rm{V}}}_{{\rm{susp}}{\rm{RBCs}}}\times {{\rm{Ht}}}_{{\rm{susp}}{\rm{RBCs}}})$$
2. The relative efficiency of enzyme inclusion (R), which shows the percentage of the specific enzyme activity included in the RBCs, relative to the maximum specific activity that can be included under given conditions (i.e. at given Ht and initial enzyme activity in the suspension):
  7 $${\rm{R}}( \% )={{\rm{A}}}_{{\rm{susp}}{\rm{EBRs}}}\times {\rm{100}}/{(A}_{{\rm{susp}}{\rm{RBCs}}}\times {{\rm{Ht}}}_{{\rm{susp}}{\rm{EBRs}}})$$
3. Absolute yield of encapsulation (also separately for each enzyme) (E), which shows how much enzyme activity (in % of the total amount added to the system), was incorporated into bioreactors:

8 $${\rm{E}}( \% )={{\rm{A}}}_{{\rm{susp}}{\rm{EBRs}}}{\times {\rm{V}}}_{{\rm{susp}}{\rm{EBRs}}}\times {\rm{100}}/{(A}_{{\rm{susp}}{\rm{RBCs}}}\times {{\rm{V}}}_{{\rm{susp}}{\rm{RBCs}}})$$

In all these formulas, V_susp RBCs_ and V_susp EBRs_ are the volumes of the suspension of the original RBCs and the EBRs obtained, respectively; A_susp RBCs_ and A_susp EBRs_ are the enzyme activities in these suspensions, and Ht_susp RBCs_ and Ht_susp EBRs_ are haematocrites of these suspensions.

### Measurement of ammonium consumption in an *in vitro* buffer containing enzymes (GDH + AAT) in free form, or as included into EBRs

The ammonium concentration in the medium was measured using an ion-selective ammonium electrode Elit (Nico 2000, Moscow, Russia) and multimeter Multitest IPL 112 (SEMIKO, Novosibirsk, Russia). The electrode was previously calibrated by successive additions to a measuring buffer of 100 mM NH_4_Cl solution to final concentrations in the range of 0.05–5 mM. The calibration buffer contained 130 mM sodium phosphate, 2.7 mM KCl, 10 mM glucose and 10 mM sodium pyruvate (pH 7.4).

When measuring the rate of ammonium consumption, the total volume of the sample in a hermetically sealed cuvette was 10 ml. The measurements were carried out at room temperature for 60–90 min at intervals of 10 s.

In experiments with EBRs containing GDH and AAT, the final enzyme activities in the cuvette averaged 0.103 ± 0.006 and 0.283 ± 0.005 IU/ml_sample_ for GDH and AAT, respectively (n = 3). A buffer solution (pH 7.4) containing 130 mM sodium phosphate, 10 mM glucose, 2.7 mM KCl, 0.2 mM α-ketoglutarate, 10 mM sodium pyruvate, 0.5 mM NH_4_Cl was placed in a measuring cuvette and supplemented by suspension of EBRs (up to the haematocrit in the sample 8–10%). In control experiments, the native erythrocytes (without the included enzymes) were used under the same conditions.

Measurement of the decrease in ammonium content in the buffer medium *in vitro* in the absence of EBRs was carried out as described above, but instead of suspension of EBRs, solutions of enzymes (GDH and AAT) were added to the medium. The final activity of GDH and AAT in the cuvette was 0.3 IU/ml and 1.5 IU/ml, respectively. NADH and pyridoxal 5′-phosphate were also added to the sample to final concentrations of 1 mM and 0.1 mM, respectively. The results were processed in the MATLAB program.

### Dependence of the stationary rate of ammonium consumption on the ratio of the AAT and GDH activities

This dependence was measured *in vitro* in a buffer solution without RBCs. The GDH activity in the solution was always 0.4 IU/ml, while the activity of AAT was 0.08, 0.2, 0.4, 0.8, 2 or 4 IU/ml. This corresponded to the ratios of AAT/GDH activities equal to 0.2, 1, 2, 5 and 10.

The measuring solution contained, besides the enzymes, 130 mM of sodium phosphate buffer (pH 7.4), 10 mM glucose, 2.7 mM KCl, 3 mM sodium pyruvate, 3 mM NADH, 0.1 mM pyridoxal 5′-phosphate, 0.1 mM α-ketoglutarate and 3 mM NH_4_Cl. The ammonium concentration was measured with the ion-selective ammonium electrode, as described above. The steady-state rate of ammonium utilization was estimated as the slope of the stationary section of the ammonium concentration curve in the system over time.

### Measurement of GDH and AAT activity

To measure the activity of enzymes encapsulated in erythrocytes, the cells were lysed by adding distilled water to the sample at a ratio 1:49. The activity of GDH and AAT in lysates was measured spectrophotometrically on a Biochrom^®^ Anthos Zenyth 340rt microplate reader (Biochrom, Ltd., Cambridge, UK) at λ = 340 nm.

The GDH activity was measured according to the method of McCarthy and Tipton^38^ with some modifications. Lysate of EBRs (10 μl) was added to 320 μl of 50 mM potassium phosphate buffer (pH 7.4) containing 0.16 mM NADH, and the mixture was incubated 10 min at 30 °C. Then, 10 μl of a 3.4 M solution of NH_4_Cl was added to each well of the plate and the reaction was started by adding a 0.8 M solution of α-ketoglutarate to a final concentration of 5 mM. The final volume of the mixture in the well was 340 μl.

The AAT activity was measured according to the procedure described by Bergmeyer H.-U. *et al*.^39^. A reaction mixture containing 100 mM TRIS buffer (pH 7.4), 500 mM alanine, 0.1 mM pyridoxal 5′-phosphate, 0.18 mM NADH and 1.2 IU/ml lactate dehydrogenase (LDH) was incubated for 10 min at 30 °C. The reaction was started by the addition of α-ketoglutarate to a final concentration of 15 mM. The total volume of the reaction mixture in the measuring well was 340 μl.

### Model of hyperammoniemia in mice

The functional activity of EBRs with GDH and AAT was tested *in vivo* in a model of induced hyperammoniemia in mice. Male mice of Swiss line (weight 25–30 g) were used. Ammocytes were obtained by the method described above (dialysis in bags) from a mixture of the blood of a part of animals obtained by decapitation. The concentrations of GDH and AAT when loaded into red blood cells were 10 IU/ml of suspension (final Ht 65%). Dialysis was carried out for 2 hours at osmolality of the hypoosmotic buffer 60–65 mOsm/kg and 4 °C. The procedures for resealing and washing the resulting EBRs were standard. It was shown that the percentage of incorporation of both enzymes was insignificant. The average specific activity amounted (mean ± SEM) 1.313 ± 0.106 IU/ml_RBCs_ and 1.612 ± 0.197 IU/ml_RBCs_ for GDH and AAT, respectively (n = 12). The activity of these enzymes was not detected in the supernatant obtained after the last washing of ammocytes, and on the outside of the ammocyte membrane, which indicated that the encapsulated enzymes were inside the ammocyte. The developed method made it possible to obtain 50–70% of cells, which practically did not differ from the original native red blood cells by their biochemical parameters.

To determine the functional activity of ammocytes *in vivo*, they were diluted 2 times with physiological saline containing an additional 5 mM glucose and injected in a volume of 0.4 ml into the tail vein of mice (Swiss line) weighing 30 g. The final hematocrit of ammocytes in the blood of mice was approximately 5.4%. Immediately after this, the mice were injected with ammonium acetate at a dose of 2.5 mmol/kg, which led to a rapid increase in the blood ammonia concentration to about 1.1–1.2 mmol/l. This concentration did not cause any behavioral reactions in animals (LD_100_ for mice is 12 mmol/kg). Blood was collected from the retroorbital sinus 5, 30, 60, and 120 min after the introduction of ammonium acetate, which made it possible to determine the dynamics of changes in the concentration of ammonia in the blood of one animal. Ammonium in the samples was determined using a microfluorimetric method measuring GDH reaction, i.e. the decrease in NADH fluorescence in the reaction of α-ketoglutarate with ammonium coupled with the reaction of NADH oxidation (λ_excitation_ = 340 nm, λ_emission_ = 450 nm)^40^. As a control, animals were used, which received, instead of ammocytes, cells that passed all stages of the encapsulation procedure, but without introducing enzymes.

### Statistical analysis

The distributions of all the experimental parameters were normal (according to the D’Agostino-Pearson test (MedCalc Statistical Software bvba, version 14.12, Belgium)). All the experimental results were analysed using one–way ANOVA and presented as the means ± standard deviations (SD) or standard errors of mean (SEM). The difference was considered significant at *P* < *0.05*.

## Results

### Mathematical models of various EBRs for ammonium removal

#### General requirements for bioreactors

**Figure 1:**
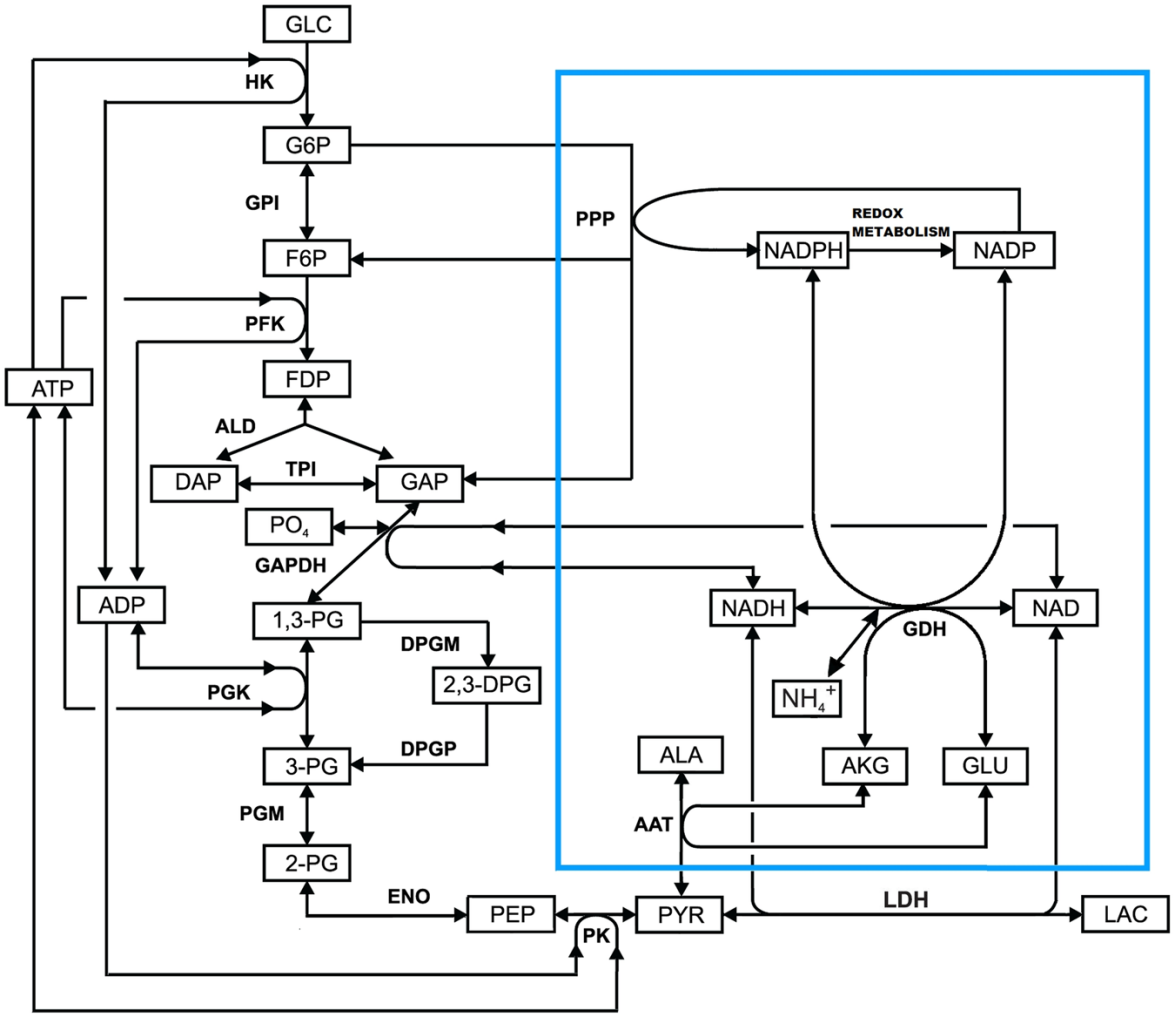
The glycolysis reactions and the reactions of included enzymes (GDH + AAT) in erythrocyte. The following abbreviations are used: GLC – glucose; G6P – glucose-6-phosphate; F6P – fructose-6-phosphate; FDP – fructose-1,6-diphosphate; DAP - dihydroxyacetone phosphate; GAP - glyceraldehyde phosphate; 1,3-DPG – 1,3-diphosphoglycerate; 2,3-DPG – 2,3 diphosphoglycerate; 3-PG – 3-phosphoglycerate; 2-PG – 2-phosphoglycerate; PEP - phosphoenolpyruvate; PYR - pyruvate; LAC - lactate; NAD – nicotinamide adenine dinucleotide; NADP – nicotinamide adenine dinucleotide phosphate; GLU – glutamic acid; AKG – α-ketoglutarate; ALA - alanine; ATP - adenosine triphosphate; ADP – adenosine diphosphate; AMP – adenosine monophosphate; PO_4_ – inorganic phosphate; HK - hexokinase; GPI – glucose-6-phosphate isomerase; PFK – phosphofructokinase; ALD - aldolase; TPI – triosephosphate isomerase; GAPDH – glyceraldehyde phosphate dehydrogenase; PGK – phosphoglycerate kinase; PGM – phosphoglycerate mutase; ENO - enolase; PK – pyruvate kinase; LDH – lactate dehydrogenase; GDH – glutamate dehydrogenase; AAT – alanine aminotransferase; PPP – pentose phosphate pathway; NH_4_^+^ - ammonium ion.

Mathematical models for four types of EBRs described above were analysed. As an example, Fig. 1 presents a full scheme of reactions in RBCs loaded with GDH and AAT (an embedded enzyme system is presented inside a blue frame). Given that cellular bioreactors should function in the body for many tens of hours and that the characteristic times of most metabolic processes in erythrocytes do not exceed several tens of minutes, it must be assumed that the created bioreactor will function under physiological conditions in a quasi-steady state, i.e., the concentrations of all intracellular metabolites of glycolysis such as glucose, LAC and PYR, will remain constant for many hours, despite the fact that the bioreactor produces or consumes these substances. Most calculations were performed under the assumption that the processes under consideration are quasi-stationary. In some calculations, we modelled the conditions when the concentrations of extracellular metabolites changed sufficiently rapidly. The results of these calculations depend on the volume and composition of the extracellular medium. These cases are specially noted in the description of the experimental conditions. The developed mathematical models were used to calculate the kinetics of metabolites of the built-in reactions and the rates of ammonium depletion in the presence of the investigated EBRs. We assumed that a constant influx of ammonium (AMM) in the circulation exists in the body. The steady-state rate of ammonium utilization (*V*_*AMM*_) was determined for all bioreactors as the slope for the stationary part of the curve of increasing concentration of utilized AMM over time (Supplemental Fig. S1).

A lower value from the experimentally determined interval (Table 2) was used for each glycolysis metabolite as the initial condition for calculating the steady state.

Stationarity of the reactor depends on the rates of the corresponding enzymatic reactions and the permeability of the RBC membrane for the substrates and products of these reactions. Hence, the efficiency of the reactor may be determined not only by the activity of the encapsulated enzymes but also by the membrane permeability for all metabolites, particularly regarding the metabolite that passes through the membrane most poorly.

Bioreactors containing various enzymes able to utilize ammonium are presented below.

#### Bioreactor based on GS (EBR # 1)

**Figure 2:**
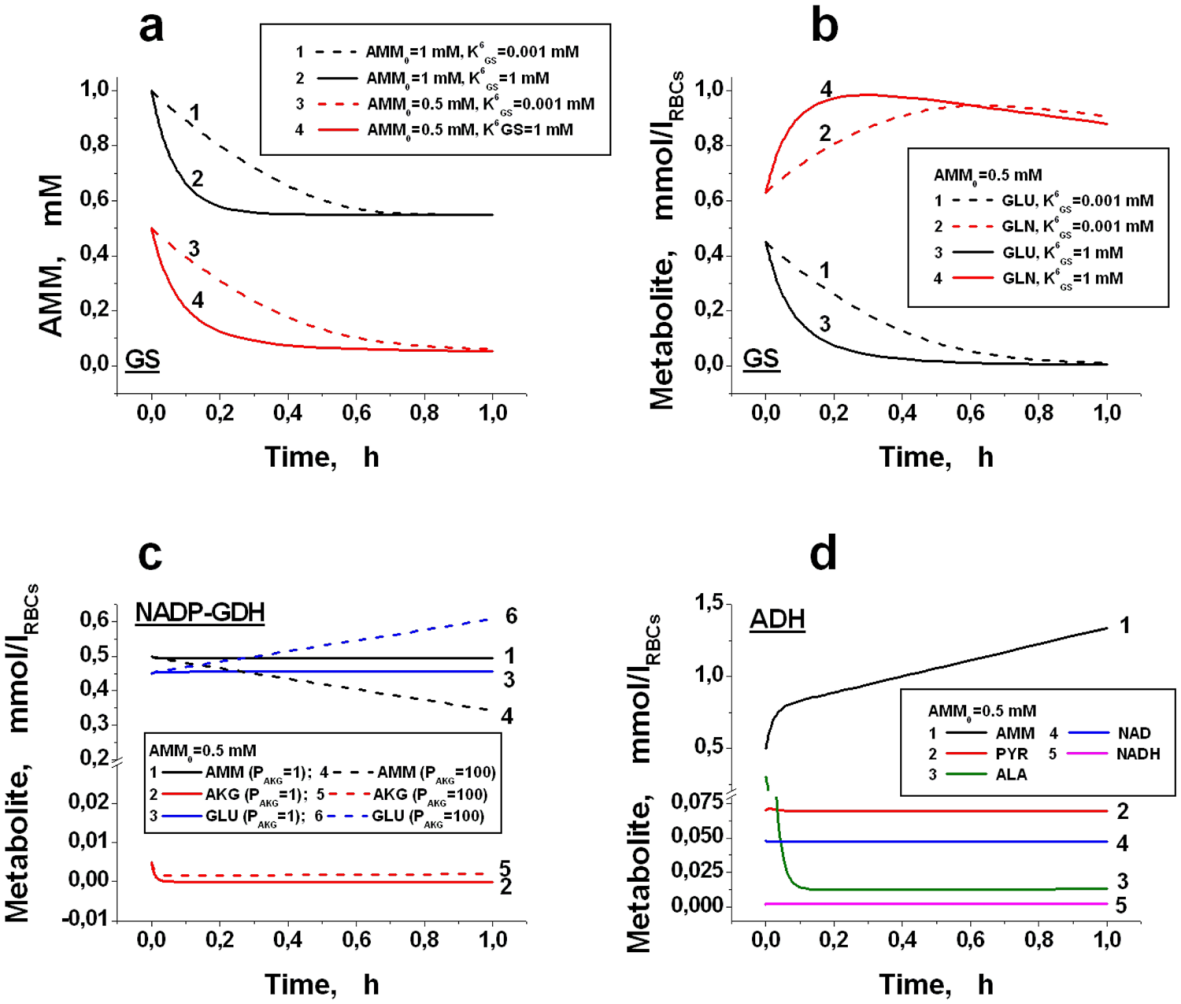
Kinetics of ammonium (AMM) and other metabolites in bioreactors loaded with various individual enzymes. The AMM concentration is not stationary and can change only due to the reaction in the bioreactor. The haematocrit of all the EBRs is 100%. (**a**) The decrease in AMM in the presence of EBRs containing GS (1 IU/ml_RBCs_). The initial АММ concentration (AMM_0_) is 1 mM (1, 2) or 0.5 mM (3, 4). The equilibrium constants K^6^_GS_ for curves (1, 3) and (2, 4) are 0.001 or 1 mM, respectively. (**b**) The kinetics of glutamic acid (GLU) and glutamine (GLN) in erythrocytes containing GS (1 IU/ml_RBCs_). AMM_0_ is 0.5 mM; the K^6^_GS_ constant values are 0.001 mM (1, 2) or 1 mM (3, 4). (**c**) The kinetics of AMM (1, 4), α-ketoglutarate (AKG) (2, 5) and GLU (3, 6) in EBRs containing NADP-dependent GDH (10 IU/ml_RBCs_). The calculation is made for normal physiological (1–3) and increased by 100 times (4–6) permeability of the RBC membrane for AKG (relative permeability for AKG (P_AKG_) is 1 or 100, respectively). (**d**) The kinetics of AMM (1), pyruvate (PYR) (2), alanine (ALA) (3), NAD (4) and NADH (5) for EBRs with ADH (1 IU/ml_RBCs_). AMM_0_ is 0.5 mM.

Under conditions that exist in normal erythrocytes, the equilibrium of the GS reaction (1) shifts in the direction of ammonium consumption (K_eq_ ~1.2 × 10^3^)^41^. Because the reaction substrate (GLU) cannot pass through the RBC membrane^28^, the reaction will proceed until the complete depletion of GLU inside the cell, and then will stop (Fig. 2a).

The value of the constant for ammonium dissociation from the GS-substrate complex (K^6^_GS_, Supplemental Table S8) was not found in the literature. However, the calculation showed that the value of this constant does not essentially affect the kinetics of ammonium depletion and practically does not affect its final level in the system (under certain levels of GLU, AKG and the initial concentration of AMM). This is confirmed by calculations shown in Fig. 2a,b, where the kinetic curves for various metabolites of the GS reaction (1) calculated for K^6^_GS_ values, differing 1000-fold are presented. In these calculations, it was assumed that the plasma did not have a constant AMM influx, i.e., the plasma contained some initial concentration of AMM, which was then changed only by the reaction taking place in the bioreactor. The final ammonium level achieved in plasma is determined by the complete depletion of GLU in the cells and depends on the initial AMM concentration. This assumption is clearly confirmed in Fig. 2a, which shows the plasma ammonium decrease curves calculated at a GLU concentration of 0.45 mM and with initial AMM concentrations of 1 mM and 0.5 mM. The final concentrations of ammonium under these conditions are 0.55 and 0.05 mM, respectively. Thus, in the absence of a constant supply of ammonium to the system, the achieved decrease in ammonium concentration is not determined by the establishment of a steady state. This decrease is virtually equal to the initial concentration of GLU. This finding confirms that the reaction proceeds until this metabolite is completely depleted. Hence, *in vivo* in patients with hyperammonaemia, when a constant influx of ammonium in the circulation exists, a glutamine synthetase-based reactor cannot continuously decrease the concentration of ammonium to its normal level.

#### Bioreactor based on GDH (EBR # 2)

The equilibrium constant for the GDH reaction (in the ammonium binding direction) for NADP-dependent GDH under physiological conditions^42^ is 5.3 × 10^14^ M^−1^. At the same time, the corresponding effective masses ratio for this reaction under physiological conditions is equal to:

$$[{\rm{GLU}}]\times [{\rm{NADP}}]\times [{{\rm{H}}}_{2}{\rm{O}}]/[{\rm{AKG}}]\times [{\rm{NADPH}}]\times [{{\rm{NH}}}_{4}^{+}]\times [{{\rm{H}}}^{+}]={\rm{1.9}}\times {{\rm{10}}}^{{\rm{13}}}{{\rm{M}}}^{-{\rm{1}}}$$

This ratio is approximately 28 times lower than the equilibrium constant. Under these conditions, the reaction will proceed in the direction of ammonium binding until the effective masses ratio becomes equal to the equilibrium constant. Inefficiency of the system, loaded with only GDH, is caused by insufficiently high permeability of the cell membrane for AKG. Figure 2c shows the kinetics of AKG in the bioreactor. In normal RBCs, the concentration of AKG is rather low (~0.005 mmol/l_RBCs_), and this metabolite passes slowly through the RBC membrane^28,29^ (coefficient of permeability: 0.029 h^−1^ (Table 1)). Thus, after the appearance of ammonium, the concentration of AKG decreases during 0.1 h to almost 0 (Fig. 2c). Hence, a very low stationary rate of ammonium removal is quickly achieved, which is limited by the slow supply of AKG into the cell from the plasma (Fig. 2c, curve 1). This observation is confirmed by the fact that a 100-fold increase in the membrane permeability for AKG proportionally increases the rate of ammonium removal from 0.0016 to ~0.16 mmol/h × l_RBCs_ (Supplemental Fig. S2, the insert). However, this approach cannot essentially improve the efficiency of bioreactors with GDH considering that in practice, we cannot significantly change the permeability of the RBC membrane for AKG (Supplemental Figs S2–S4).

#### Bioreactor based on ADH (EBR # 3)

For the bioreactor with ADH, the calculations showed that at normal intracellular and extracellular physiological concentrations of all metabolites^28,32,43,44^, such a system would produce rather than remove ammonium (Fig. 2d). The equilibrium constant of the ADH reaction (in the direction of ammonium synthesis) is equal to 3.1 × 10^−14^ M^45^. It follows that the equilibrium constant of the system in the direction of ammonium utilization is 3.2 × 10^13^ M^−1^. An estimate of the corresponding effective masses ratio within the erythrocyte, calculated for physiological conditions (Tables 1 and 2), gives the following value:

$$[{\rm{A}}{\rm{L}}{\rm{A}}]\times [{\rm{N}}{\rm{A}}{\rm{D}}]\times [{{\rm{H}}}_{2}{\rm{O}}]/[{\rm{P}}{\rm{Y}}{\rm{R}}]\times [{\rm{N}}{\rm{A}}{\rm{D}}{\rm{H}}]\times [{{\rm{N}}{\rm{H}}}_{4}^{+}]\times [{{\rm{H}}}^{+}]\approx 3.6\times {10}^{15}\,{{\rm{M}}}^{-1},$$

which is 100 times higher than the value of the equilibrium constant. Hence, under these conditions, to approach equilibrium, the reaction will proceed towards ammonium formation, which agrees with the prediction obtained from our model. A direct reaction of ammonium with pyruvate will occur only at ammonium concentrations in the plasma above 4.5 mM, that is, approximately 112.5 times higher than the plasma concentration of ammonium under normal conditions^46^.

#### Bioreactor with GDH and AAT (EBR # 4)

The disadvantages of the GDH-based bioreactor are mainly associated with the poor membrane permeability for the substrate (AKG)^29,32^ and the product (GLU)^32^ of this reaction. However, the situation may be improved if the enzyme AAT is also included in RBCs. Collectively the process of operation of these two enzymes is described by the reaction (9), which is the sum of the reactions (2) and (4):

9 $${{\rm{NH}}}_{4}^{+}+{{\rm{H}}}^{+}+{\rm{NADPH}}({\rm{or}}\,{\rm{NADH}})+{\rm{PYR}}\leftrightarrow {\rm{NADP}}({\rm{or}}\,{\rm{NAD}})+{\rm{ALA}}+{{\rm{H}}}_{2}{\rm{O}}$$

This bioreactor catalyses the formation of alanine from pyruvate and ammonium. Both these metabolites pass well through the RBC membrane^32,33^. The rate of NADPH production in glycolysis within erythrocytes is also sufficiently high, reaching 2–4 mmol/h × l_RBCs_^47^.

Thus, due to the presence of the AAT reaction, the limitations related to poor RBC membrane permeability for GLU and AKG in this system might be eliminated. GLU formed in the GDH reaction is the substrate of the AAT reaction, and its product (AKG) is necessary for the GDH reaction (see reactions (2) and (4)). As a result, a bioreactor including GDH, AAT and the erythrocyte glycolysis reactions may be able, in principle, in the presence of NH_4_^+^ to convert PYR to ALA with simultaneous oxidising of NADH (or NADPH), according to equation (9) (see above).

**Figure 3:**
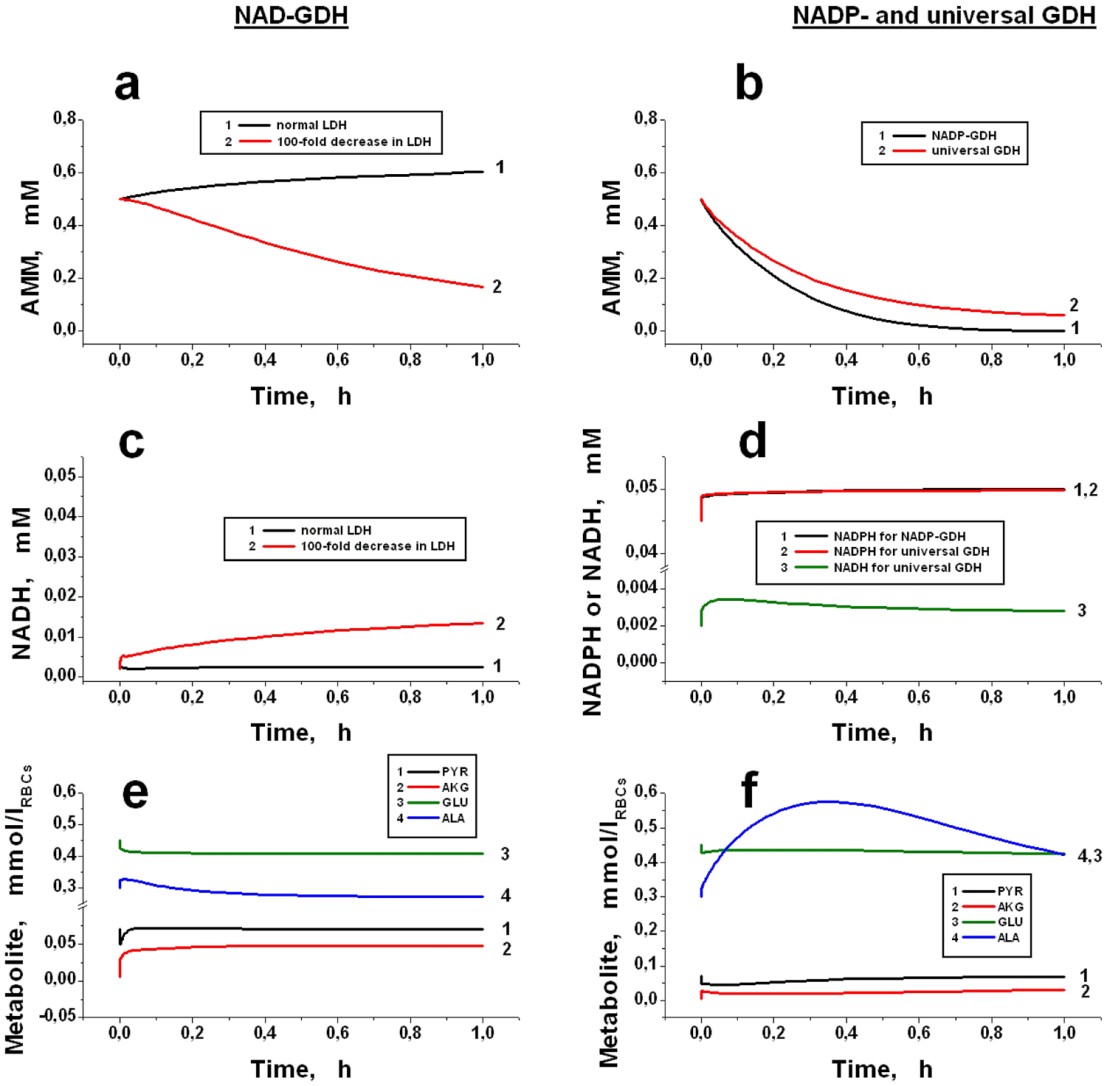
Bioreactors with jointly encapsulated AAT (50 IU/ml_RBCs_) and GDH (10 IU/ml_RBCs_) of different types: NAD-dependent (**a**,**c**,**e**), or NADP-dependent and universal (**b**,**d**,**f**). Calculated kinetics of ammonium concentrations (**a**,**b**), NADH and NADPH (**c**,**d**), as well as pyruvate (PYR), α-ketoglutarate (AKG), glutamate (GLU) and alanine (ALA) (**e**,**f**). The haematocrit of EBRs is 100%. The calculation was made for a situation without constant AMM supply into the plasma (i.e., the initial concentration AMM_0_ was 0.5 mM and could be changed only due to reactions occurring in the bioreactor). Universal GDH works equally with both NAD and NADP. For NAD-dependent GDH, the kinetics of AMM and NADH was also calculated for the lactate dehydrogenase (LDH) activity, which was decreased by two orders of magnitude (panel a, curve 2 and panel c, curve 2, respectively). PYR, AKG, GLU and ALA were calculated for ammocytes containing NAD-GDH (**e**) or NADP-GDH (**f**). The initial concentrations of each metabolite corresponded to the values in Tables 1 and 2.

The membrane permeability coefficients for pyruvate^32^ (51.8 h^−1^) and alanine^34^ (1.95 h^−1^) are rather high. This observation indicates that the restrictions on the trans-membrane fluxes of these metabolites may be shifted in such reactors in the direction of sufficiently high rates of ammonium removal. Reactions, catalysed by encapsulated enzymes, are readily reversible; thus, not only the sum reaction rate but also its direction should depend on the substrate and product concentrations in the bioreactors. The calculations showed that the most important parameter that determined the kinetics of the process was the ratio of NAD/NADH (or NADP/NADPH). The kinetics of various metabolites involved in reactions catalysed by two enzymes (AAT and GDH (NAD-, NADP- or simultaneously both NAD- and NADP-dependent)) is shown in Fig. 3. The possibility of consuming AMM by the proposed enzymes depends on the properties of GDH used in the bioreactor. The equilibrium constant of the process described by equation (9) (in the direction of ammonium consumption) is equal to the product of the corresponding constants for the GDH and AAT reactions (K_eq_ = 5.3 × 10^14^ × 0.45 M^−1^ = 2.4 × 10^14^ M^−1^).

In Fig. 3a (curve 1), the kinetics of ammonium is shown in the case when NAD-dependent GDH is used. The concentrations of PYR, LAC and other metabolites of glycolysis are within physiological values. The initial concentration of ammonium is 0.5 mM and may vary only due to the reactions occurring in the bioreactor. For this enzyme, the reaction proceeds in the direction of ammonium formation. This result obtains because the ratio of effective masses of reaction (9) under conditions existing in RBCs is equal to:

$$[{\rm{ALA}}]\times [{\rm{NAD}}]\times [{{\rm{H}}}_{2}{\rm{O}}]/[{{\rm{NH}}}_{4}^{+}]\times [{{\rm{H}}}^{+}]\times [{\rm{NADH}}]\times [{\rm{PYR}}]=1.5\times {10}^{14}[{\rm{NAD}}]/[\mathrm{NADH}]\,{{\rm{M}}}^{-1}$$

Thus, the reaction will proceed in the desired direction, provided the ratio of [NAD]/[NADH] is <1.6. However, in native RBCs, this ratio is significantly higher and maintains at an approximately constant level equal to 24 (Table 2) due to the high activity of lactate dehydrogenase (LDH). Under physiological conditions, this reaction is close to equilibrium, and proceeds in the direction of lactate production (10):

10 $${\rm{LDH}}:\,{\rm{PYR}}+{\rm{NADH}}+{{\rm{H}}}^{+}\leftrightarrow {\rm{LAC}}+{{\rm{NAD}}}^{+}$$

To shift the reaction (9) in the direction of ammonium consumption, it is necessary to increase the concentration of NADH. Under stationary erythrocyte metabolism, this may be executed by reducing the activity of LDH. The calculations showed that a noticeable consumption of ammonium might be obtained only with a significant decrease in LDH activity. Figure 3c (curve 2) and 3a (curve 2) show the kinetics of increasing NADH concentration and ammonium consumption, respectively, when the LDH activity is decreased 100-fold. In this case, the ratio [NAD]/[NADH] decreased to approximately 1.

**Figure 4:**
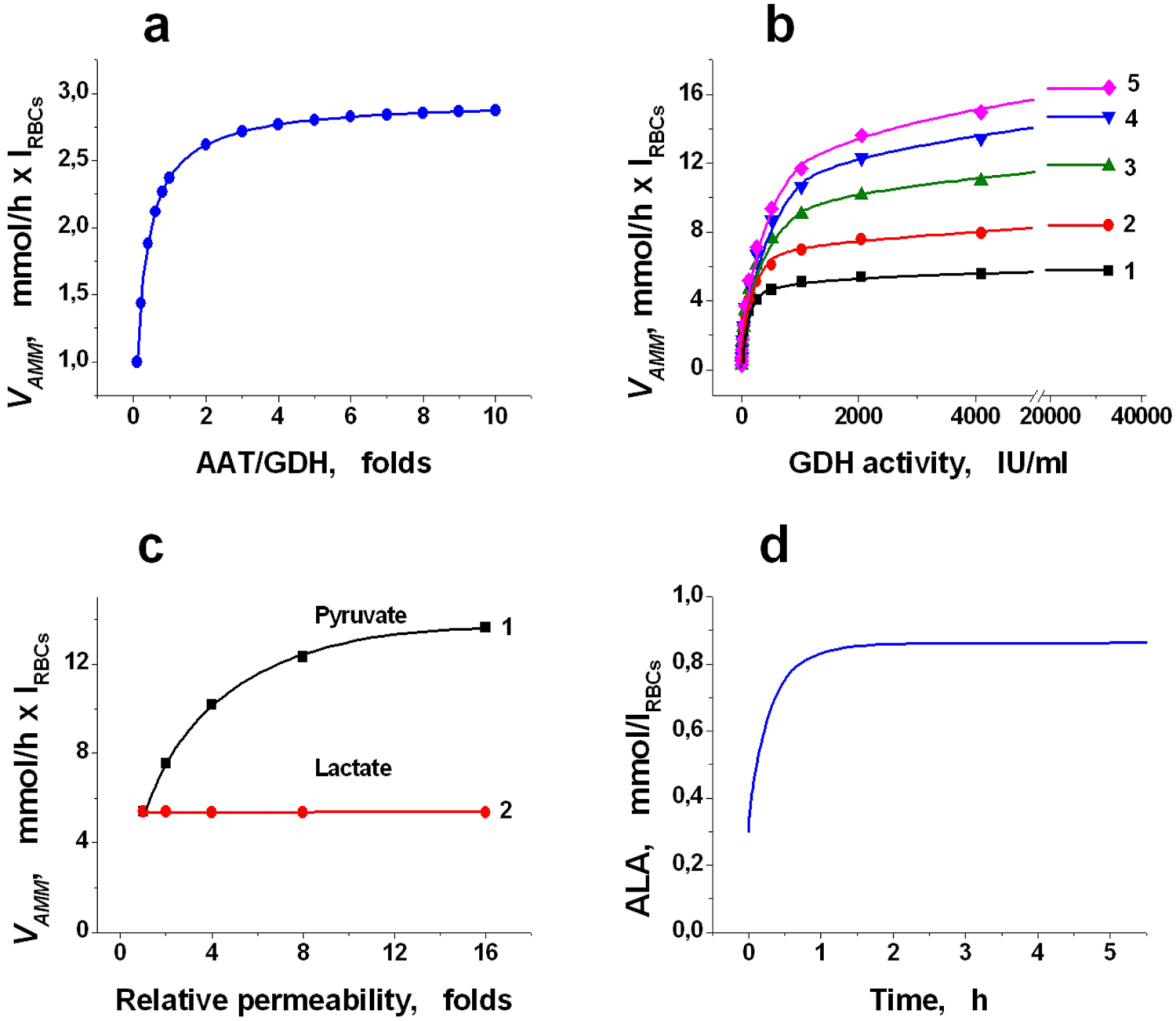
The calculated kinetic parameters of a quasi-stationary bioreactor containing GDH and AAT. The calculations were performed for a constant concentration of AMM in plasma equal to 0.5 mM. (**a**) Effect of the ratio of AAT/GDH activities on the rate of ammonium consumption (*V*_*AMM*_). GDH activity was constant and equal to 0.4 IU/ml_RBCs_, while AAT activity varied in the range of 0.04–4 IU/ml_RBCs_. (**b**) Dependence of *V*_*AMM*_ on concentrations of encapsulated enzymes (at ААТ/GDH = const = 5) at different RBC membrane permeability for pyruvate (the permeability was increased compared to physiological by 1-, 2-, 4-, 8- and 16-fold for curves 1, 2, 3, 4 and 5, respectively). (**c**) Dependence of *V*_*AMM*_ on changes in the permeability of the bioreactor membrane for pyruvate (1) or lactate (2) (for each curve, all other permeabilities are considered equal to physiological, GDH activity is 2048 IU/ml_RBCs_, and AAT/GDH activity ratio is 5). (**d**) Increase in alanine (ALA) concentration during quasi-stationary operation of a bioreactor containing GDH and AAT.

NADP-dependent GDH appears to be a much more promising enzyme for ammonium removal because in erythrocytes, the [NADPН]/[NADP] ratio is significantly greater than 1 (Table 2). In erythrocytes, NADP is reduced in the PPP and may be partially oxidised by glutathione reductase during the reduction of oxidised glutathione. The flow through PPP is usually only 4–5% of the glycolysis flow however it may be greatly increased under conditions of increased oxidation. The [NADPН]/[NADP] ratio in erythrocytes in the steady state is sufficiently large (~20)^48^. Since the equilibrium constants for NAD- and NADP-dependent GDH are close^49^, this ratio leads to stable ammonium removal by NADP-dependent GDH (Fig. 3b, curve 1). Similar kinetics was demonstrated by the universal GDH (Fig. 3b, curve 2). Cofactor concentrations (NADPH and NADH) reach a steady state very rapidly (Fig. 3d). Kinetic curves for PYR, AKG, GLU and ALA in the case of NAD- or NADP-dependent GDH are presented in Fig. 3e,f, respectively. In the case of NAD-dependent GDH, when AMM removal does not occur, the concentrations of all these metabolites almost do not change with time. For NADP-dependent GDH, AMM removal occurs (Fig. 3b), AKG, PYR and GLU concentrations reach a steady state very quickly and then do not change, and the concentration of ALA gradually increases until existing AMM is completely removed and then gradually returns to its stationary physiological level. Calculations show that the alanine concentration inside the cells cannot markedly increase even when there is a stationary ammonium influx into the system). The steady-state level for this concentration is approximately 0.86 mmol/l_RBCs_ (Fig. 4d). Thus, the obtained results confirm our assumption that it is possible to avoid the depletion of AKG and the accumulation of GLU and ALA in the bioreactor, provided GDH and AAT are included in the RBCs together.

#### Dependence of the rate of ammonium removal on the AAT/GDH ratio

The calculations of this section were performed for NADP-dependent GDH under the condition of a constant (stationary) concentration of ammonium in plasma equal to 0.5 mM. Under this condition, the system achieves a steady rate of ammonium consumption (Supplemental Fig. S1). The GDH activity in cells in all calculations was constant and equal to 0.4 IU/ml_RBCs_. The activity of AAT varied from 0.04 to 4 IU/ml_RBCs_. The haematocrit of the suspension was considered as 100%. The steady-state rate of ammonium removal increased with the increase in AAT/GDH ratio up to 4–5 and then almost stopped changing (Fig. 4a). Thus, when enzymes are encapsulated in erythrocytes, there is no reason to create an excess of AAT activity over GDH more than 5-fold.

#### Dependence of the rate of ammonium removal on RBC membrane permeability for lactate and pyruvate

The steady-state rate of ammonium consumption in plasma can be achieved only if ammonium is not only permanently removed but also enters the system. Hence, an estimate of the dependence of stationary *V*_*AMM*_ in the presence of EBRs containing GDH and AAT on the RBC membrane permeability for pyruvate and lactate was performed, assuming that the concentration of ammonium in the system was constant and equal to 0.5 mM. The AAT/GDH ratio was also considered constant and equal to 5. Other initial conditions corresponded to the values in Tables 1 and 2. The results are shown in Fig. 4b,c. Under physiological permeability of the erythrocyte membrane for pyruvate and lactate, the rate of ammonium depletion increases with increasing concentrations of the enzymes included in the EBRs (Fig. 4b, curve 1). However, starting with the activity of GDH equal to approximately 1000 IU/ml_RBCs_ the curve reaches saturation. To investigate what limits the maximum rate of ammonium removal, we calculated the same dependence, consistently increasing the membrane permeability for pyruvate by 2, 4, 8 and 16 times relative to the physiological level (Fig. 4b, curves 2–5, respectively). The achieved maximum steady-state *V*_*AMM*_ increased with increasing membrane permeability for pyruvate, though not proportionally to this increasing permeability (a 16-fold increase in permeability caused a maximum *V*_*AMM*_ increase of less than 3-fold) and eventually reached saturation (Fig. 4c, curve 1). The GDH activity (and the corresponding AAT activity), at which the curve reached saturation, also increased, though not more than 1.5–2 times. The permeability of the erythrocyte membrane for PYR (or LAC) under normal physiological conditions (Table 1) was accepted as the unit of permeability under calculating the relative RBC membrane permeability for these metabolites. A similar analysis showed that the membrane permeability for lactate did not affect the stationary rate of ammonium removal (Fig. 4c, curve 2).

Thus, even with very high activity of the enzymes encapsulated in EBRs, the equilibrium of the LDH reaction does not shift towards pyruvate production. The reaction direction is determined only by the ratio of NAD/NADH concentrations, which does not change with a change in the permeability of the erythrocyte membrane for pyruvate and lactate. According to the model, the maximum rate of ammonium removal under the physiological permeability of the erythrocyte membrane for lactate and pyruvate is approximately 6 mmol/h × l_RBCs_, and an increase in permeability for pyruvate by 10–15 times leads to an increase in this rate of approximately 2–2.5-fold (Fig. 4c).

### Experimental study of the incorporation of GDH and AAT into human erythrocytes

#### Encapsulation efficiency

**Figure 5:**
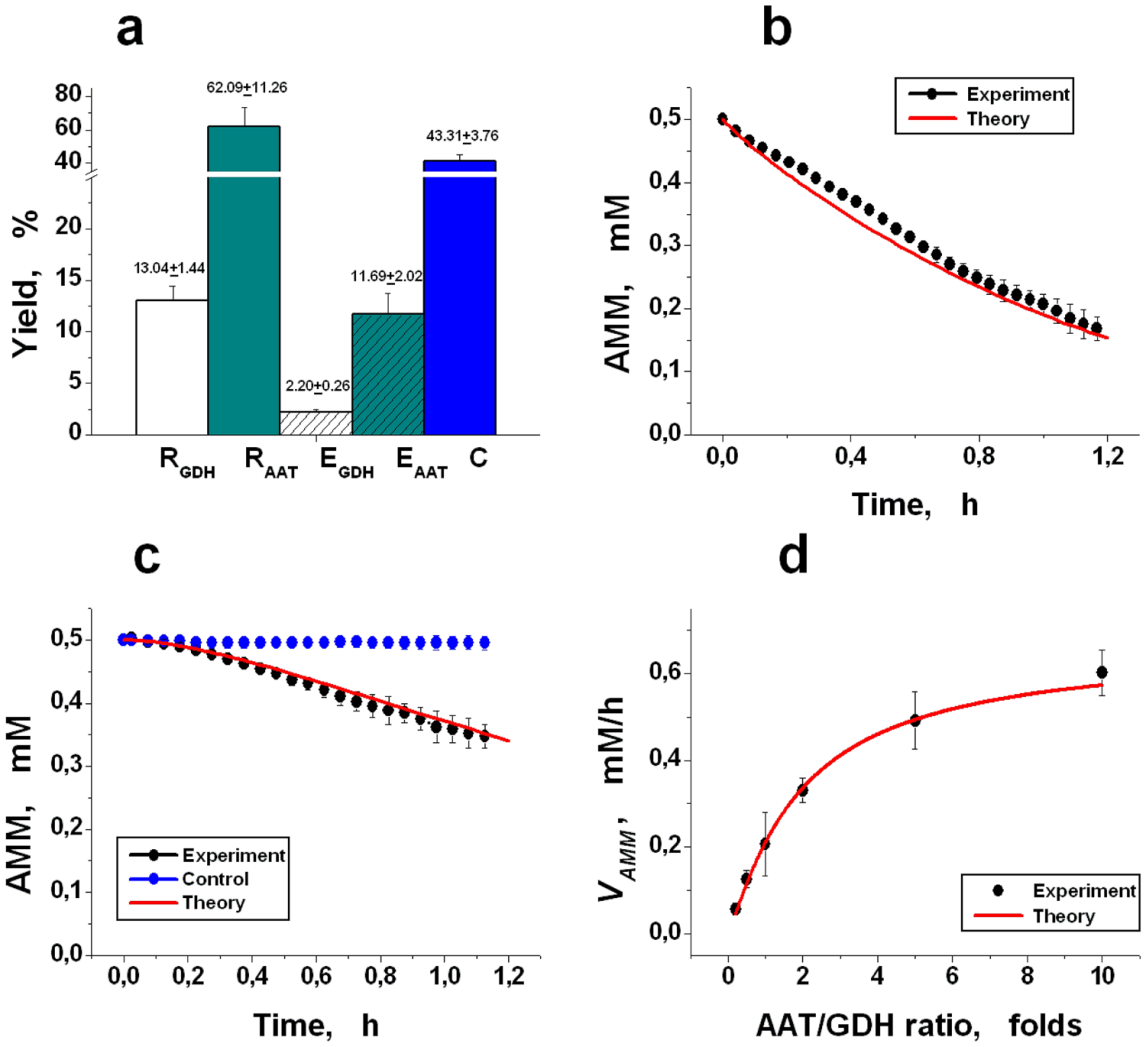
The efficiency of GDH and AAT encapsulation into erythrocytes and comparison of experimental results and model calculations for systems containing these enzymes. (**a**) The relative inclusion efficiency (R) and the yield of encapsulation (E) are presented for each enzyme. Additionally, the yield of the cells (C) is shown. The GDH and AAT concentrations in the initial suspension of RBCs during loading were 10 IU/ml_suspension_ and 5 IU/ml_suspension_, respectively. All the parameters were normally distributed. The mean values ± SEM (n = 10) are presented. (**b**) Comparison of the theoretically calculated and *in vitro* measured rate of ammonium utilization by a mixture of GDH and AAT directly added to a buffer containing 0.5 mM ammonium. The final activities of GDH and AAT in the mixture were 0.3 IU/ml and 1.5 IU/ml, respectively. The mean values ± SD are shown (n = 4). (**c**) The experimental and theoretically calculated rates of ammonium consumption by EBRs containing GDH and AAT. The final enzyme activities were 0.10 and 0.28 IU/ml_suspension_, respectively (the haematocrit of the suspension averaged 9.3%). As a control the RBCs without included enzymes were used (n = 4). The mean values ± SD are shown (n = 3). (**d**) Comparison of the calculated and experimentally measured stationary rate of ammonium consumption (in a medium without erythrocytes) at the different ratios of AAT and GDH activities. The values for the mean ± SD are presented (n = 4). The conditions for measuring and the concentrations of the metabolites for modelling are described in the Methods section and shown in Tables 1 and 2.

The joint encapsulation of GDH and AAT in erythrocytes of different donors was performed by the hypotonic dialysis-resealing method (Methods). The efficiency of the procedure was evaluated by three parameters: cell yield (C) and for each enzyme, the relative inclusion efficiency (R), and the encapsulation yield (E) (Methods, formulae 6–8, respectively). The results are shown in Fig. 5a. The yield of the cells was 43.31 ± 3.76% (n = 10). The R and E yields of AAT encapsulation were significantly higher than the corresponding yields for GDH (one-way ANOVA (two-tailed), *P* < 0.05). This result agrees well with previously published studies and is explained by the fact that GDH molecules have a large molecular weight (340 kDa) and can aggregate with the formation of molecular aggregates of large size under an increase in the concentration of the GDH protein above 0.1–0.3 mg/ml^26,50^. AAT molecules have approximately 3 times less molecular weight (115 kDa)^51^ and are not able to aggregate.

#### Comparison of the experimental and theoretically calculated rate of ammonium removal *in vitro*

The mathematically simulated rate of ammonium consumption was compared with the experimental results measured *in vitro* for two enzymes (GDH + AAT), added to the buffer solution either directly or inside the human EBRs. The results are shown in Fig. 5. The red curves represent the mathematical simulation results, and the black curves are the averaged results obtained in the experiments (mean ± SD). For enzymes added directly to the buffer solution (GDH of 0.3 IU/ml and AAT of 1.5 IU/ml) at an initial ammonium concentration in the buffer equal to 0.5 mM, the measured ammonium removal rate (0.39 mM/h) correlated with the calculated rate (0.42 mM/h) within 10% (n = 4) (Fig. 5b). For EBRs, the rate of ammonium consumption was measured in a suspension with a haematocrit of 8–10%. The ammonium removal rate measured in the experiments (mean ± SD, n = 3) was in satisfactory agreement with the theoretical rate and after recalculation to a haematocrit of 100% was equal to 1.5 mmol/h × l_RBCs_ (Fig. 5c). The control erythrocytes under these conditions did not change the ammonium level over the entire time of the experiments (Fig. 5c, blue curve, n = 4). Figure 5d shows the calculated and experimental stationary ammonium removal rate in a buffer without erythrocytes depending on the AAT/GDH ratio in the system (mean ± SD, n = 4). The measured values coincided well with the values predicted theoretically for the entire range of the studied AAT/GDH ratios.

In addition to the described characteristics, other properties of new bioreactors, such as osmotic resistance, standard erythrocyte indices and changes in the activity of the included enzymes and of the other parameters listed above, during storage of the bioreactors for a week at 4 °C, were also studied. These experiments showed that the new human EBRs were well preserved during one week of storage. Due to the article size limitations, these results are detailed in Supplementary Information (Figs S5 and S6).

### Ammonium removal *in vivo* in a model of hyperammoniemia in mice

**Figure 6:**
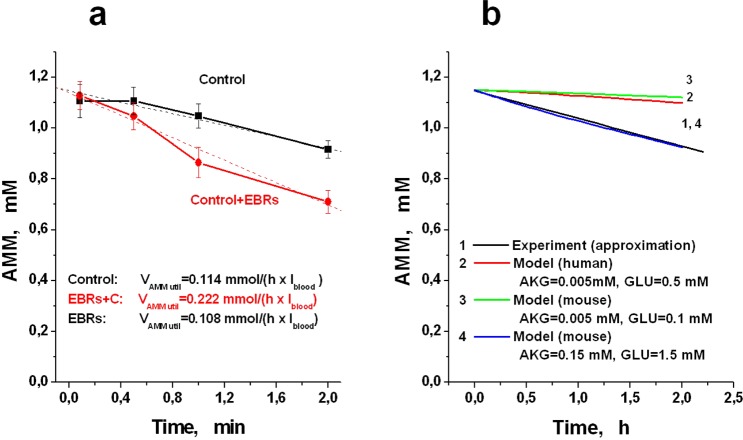
Model of hyperammoniemia in mice *in vivo*. (**a**) Decrease of ammonia in the blood of mice (Swiss line, male, weight 30 g) with hyperammonemia, which was induced by one injection into the tail vein of ammonium acetate at a dose of 2.5 mmol/kg. Each animal (n = 12) received 135 µl of ERBs, containing glutamate dehydrogenase and alanine aminotransferase (1.313 ± 0.106 and 1.612 ± 0.197 IU/ml_RBCs_ for GDH and AAT, respectively) in normal saline (total volume 0.4 ml) (a curve EBRs + C). The final hematocrit of EBRs in the blood of mice was approximately 5.4%. As a control, animals were used, which received, instead of ammocytes, cells that passed all stages of the encapsulation procedure, but without introducing enzymes (n = 12) (curve Control). (**b**) Comparison of experimental curve of AMM decreasing in mice (1) with curves calculated using a mathematical models of EBRs: (2) – a model for human erythrocytes (concentrations of AKG and GLU were set as in human RBCs (0.005 and 0.5 mmol/l_RBCs_, respectively)); (3) – a mouse model with 0.005 and 0.1 mmol/l_RBCs_, for AKG and GLU, respectively; (4) – a mouse model with the increased AKG and GLU concentrations (0.15 mmol/l_RBCs_ and 1.5 mmol/l_RBCs_, respectively).

The functional efficacy of EBR containing GDH and AAT was also measured *in vivo* in a mouse hyperammonemia model. For this, mouse erythrocyte-bioreactors were prepared. The results are presented in Fig. 6. Since healthy animals were used, most of the introduced ammonium acetate was quickly detoxified by their healthy liver even in the absence of introduced EBRs (see Control curve in Fig. 6a). However, the rate of ammonium utilization in the presence of bioreactors is almost 2 times higher than in control animals. In addition, the bioreactors continued to work even 2 h after the administration, which distinguished them from the bioreactors with GS or GDH, described earlier in the literature^23,25^. The rate of ammonium utilization, calculated according to the data obtained, was equal to 2.0 mmol/h × 1_EBRs_. To compare this value with a model calculation, we first used a model developed for human EBRs, and then introduced constants and concentrations into this model, known for some glycolysis reactions in mice (Fig. 6b, and Supplemental Tables S9 and S10). However, we did not manage to find all the necessary parameters in the literature; therefore, the values for the corresponding glycolytic reaction in human erythrocytes were used for the missing values. Even under these conditions, the obtained experimental data are in fairly good agreement with the model calculations. By varying the values of some unknown or not reliably known parameters for mice (for example, AKG or GLU concentrations), one can bring closer the calculated and experimentally obtained rates of ammonia utilization (Fig. 6b).

## Discussion

It is known, that RBCs can be carriers for some drugs, enzymes included. Significant advantages of enzymes within erythrocytes in comparison with the traditional forms of these preparations were already described above. The very idea to use erythrocytes as bioreactors for the inclusion of enzymes has been known for a long time, but so far it had been successfully implemented in the clinic only for the antitumor drug L-asparaginase. The reasons for the unsuccessful attempts have not been practically analysed. In this study we for the first time perform a fundamental theoretical analysis of the factors affecting the effectiveness of new medicinal forms of enzyme preparations based on erythrocytes as carriers.

The theoretical analysis of various systems with enzymes capable of using ammonium showed that the efficiency of these EBRs is limited not only by the activity of the encapsulated enzymes, but, first of all, by the permeability of the RBC membrane for the substrates and products of these reactions. Under physiological conditions, only EBRs with two jointly included enzymes (GDH and AAT) could efficiently consume ammonium for a long period. This was possible due to the effective conversion of GLU formed in the GDH reaction into AKG (in the AAT reaction) and the repeated participation of this AKG in the GDH-catalysed reaction. In this system, the challenge of low membrane permeability for α-ketoglutarate and glutamate was solved by creating a metabolic pathway whereby α-ketoglutarate and L-glutamic acid were produced and consumed in a cyclical process; hence, the system became independent of their transport. However, in this system, ammonium reacted with pyruvate. According to our theoretical estimates, under physiological conditions, the maximum rate of ammonium removal using EBRs containing GDH and AAT should be limited only by the supply of pyruvate into RBCs (~6 mmol/h × l_RBCs_ under physiological conditions). The permeability coefficient for the product of this reaction, alanine, is quite high (~1.95 h^−1^), that prevents the accumulation of this amino acid within the cells in high concentrations.

The conclusions of the theoretical study were verified experimentally. GDH and AAT were successfully encapsulated into erythrocytes by the hypoosmotic dialysis method. Obtained EBRs had a satisfactory survival rate. The activity of enzymes within the EBRs and their haematological indices were well preserved during one week of storage. The shape of the osmotic fragility curve for EBRs was changed compared to original RBCs on day 0, but was restored as a result of normal metabolism of bioreactors after 6 days of storage in a special solution containing adenine and glucose. Measurement of the rate of ammonium removal in buffer in the presence of a mixture of free enzymes (GDH + AAT) or in a suspension of EBRs obtained showed, that the model described the experiments well. The dependence of this rate on the AAT/GDH ratio (in a cell-free medium) also coincided with the theoretically calculated one. Thus, the experimental results confirmed the correctness of the proposed mathematical models and conclusions, formulated on their basis. They also showed the opportunity for effective ammonium removal *in vitro* using new EBRs. The developed approach is very promising for the analysis of the efficiency of any enzymes encapsulated in erythrocytes.

The functional efficacy of EBRs with GDH and AAT was also confirmed *in vivo* in an experimental model of induced hyperammoniemia in mice. As expected, these EBRs worked for a longer time compare to previously described bioreactors. Unfortunately, the total time of the *in vivo* experiment was restricted only 2 h (due to the large volume of necessary blood samples from one animal). This is a limitation of our work, but we suppose that it does not change the main findings. A model of mouse bioreactors, in which reaction constants and concentrations of metabolites known for the mouse were used, described the obtained ammonium consumption rate in principle. The observed discrepancies can be explained by the lack of a part of the necessary parameters, which we took from the human EBR model.

Thus, it was proved that EBRs loaded with GDH and AAT are able to work in the body.

Usually, a blood transfusion is not used as a treatment strategy in the presence of a deficiency of urea cycle enzymes or in liver diseases. However, we believe that the return to the patient in small quantities (up to 100–200 ml) of his own or donor’s erythrocytes, which have undergone the enzyme incorporation procedure, cannot be a limiting factor of therapy. The question about the possibility of ammocytes practical application is very important. It should be noted, that the aforementioned maximum rate of ammonium removal with ammocytes hardly can be achieved in practice as far from all erythrocytes in the patient’s blood are bioreactors; moreover, the activity of bovine GDH encapsulated into erythrocytes is rather low because of the high molecular weight of this enzyme and its ability to aggregate.

To increase the efficiency of EBRs, GDH from other biological sources may be used. Potential glutamate dehydrogenases to replace bovine liver GDH must meet three basic requirements: (1) NADP-specificity or ability to use both NADP and NAD; (2) Minimal possible molecular weight; and (3) Maximal possible specific activity. Among bacteria and archaea, glutamate dehydrogenases exist that approximately satisfy all these requirements^52^. Their isolation and encapsulation into RBCs is the subject of our further research.

More effective methods for enzymes encapsulation (e.g., a method of dialysis in the flow) and other types of GDH with higher specific activity (such as GDH from *Proteus sp*., which have activity regulation very resembling regulation of bovine enzyme, but cannot aggregate) may allow one to increase the cell yield and the specific activity of GDH inside the RBCs. If the indicated parameters will be increased to 60% and 50 and more IU/ml_RBCs_, correspondingly, about 5000 IU could be administered to a patient in 100 ml of EBRs. Such a dose of GDH (5000 IU/5 l_blood_) could decrease the ammonium concentration in the blood at the rate of 1000 µM/min (or 60 mM/h). This is a very high rate compared to 600 µM/day for the best drugs in Supplemental Table S11. However, such a rate can be achieved only under conditions of complete saturation of enzymes with their substrates. In real RBCs this rate is limited by the pyruvate and AKG concentrations. The maximum rate of ammonium utilization by our EBRs, calculated theoretically, is 6 mmol/h × l_EBRs_ (at hematocrit 100%). We are going to transfuse a patient 100–200 ml of EBRs. Therefore, the maximum rate we could receive after administration of 200 ml of EBRs in the body with a blood volume of 5 liters should be 1.2 mmol/h × 5 l_blood_ = 0.24 mM/h, or 5.76 mM/day. This value is more than 10 times more than similar values for other medications (see Supplemental Table S11). Currently, we cannot accurately predict the dose that should be given to different patients with hyperammonemia, since the ammonium concentration in these patients may vary (from 0.1 to 4 mM). This problem is now under investigation, but we can expect that EBRs are promising in the treatment of hyperammonemia.

## Supplementary information

Supplementary Information

## Data Availability

All data generated or analysed during this study are included in this published article (and its Supplementary Information File).
