# Supplementary material for: Erythrocytes as bioreactors to decrease excess ammonium concentration in blood
Source: Sci Rep. 2019 Feb 6;9:1455. doi: 10.1038/s41598-018-37828-5 (PMC6365525; doi:10.1038/s41598-018-37828-5)
Supplement: Supplementary file 1 — Supplementary Information [file 41598_2018_37828_MOESM1_ESM.pdf]

Erythrocytes as bioreactors to decrease excess ammonium concentration in blood

Eugeniy S. Protasov, Daria V. Borsakova, Yuliya G. Alexandrovich,
Anatoliy V. Korotkov, Elena A. Kosenko, Andrey A. Butylin, Fazoil I. Ataullakhanov,
Elena I. Sinauridze

SUPPLEMENTARY INFORMATION

Parameters and equations of mathematical models

The study developed several mathematical models for various included into red blood cells (erythrocytes, RBCs) enzymatic systems, which were supposed could be able to remove ammonium from the blood. The systems of differential equations for all these models had a common part that included equations for glycolysis metabolites, which did not change because of the encapsulation of additional enzymes into erythrocytes. These equations are presented in Table S1. Further, in Tables S2-S6 the residual parts of the differential equation systems are presented describing the glycolysis metabolites (for each of the models separately), which were changed as a result of the investigated enzymes encapsulation into RBCs. Tables S7 and S8 present equations for enzymes of glycolysis, pentose phosphate pathway and energy-consuming processes in RBCs, as well as equations for enzymes included into RBCs, respectively. Each *Vi* symbol presents the rate of the reaction catalysed by the enzyme *i*.

Table S1. Differential equations for the glycolysis metabolites, common for all models [a)].

Variable	Differential equation
Glucose-6-phosphate (G6P)	$\frac{d[G6P]}{dt} = V_{HK} - V_{PGI} - V_{GPDH}$
Fructose-6-phosphate (F6P)	$\frac{d[F6P]}{dt} = V_{PGI} - V_{PFK} + \frac{2}{3}V_{GPDH}$
Fructose-1,6-diphosphate (FDP)	$\frac{d[FDP]}{dt} = V_{PFK} - V_{ALD}$
Dihydroxyacetone phosphate (DAP)	$\frac{d[DAP]}{dt} = V_{ALD} - V_{TPI}$
Glyceraldehyde-3-phosphate (GAP)	$\frac{d[GAP]}{dt} = V_{ALD} + V_{TPI} + \frac{1}{3}V_{GPDH} - V_{GAPDH}$

1,3-Diphosphoglycerate (1,3DPG)	$\frac{d[1{,}3DPG]}{dt} = V_{GAPDH} - V_{DPGM} - V_{PGK}$
2,3- Diphosphoglycerate (2,3DPG)	$\frac{d[2{,}3DPG]}{dt} = V_{DPGM} - V_{DPGP}$
3- Phosphoglycerate (3PG)	$\frac{d[3PG]}{dt} = V_{PGK} + V_{DPGP} - V_{PGM}$
2-Phosphoglycerate (2PG)	$\frac{d[2PG]}{dt} = V_{PGM} - V_{ENO}$
Phosphoenolpyruvate (PEP)	$\frac{d[PEP]}{dt} = V_{ENO} - V_{PK}$
Lactate (LAC)	$\frac{d[LAC]}{dt} = V_{LDH} - V_{transpLAC}$

[a] Glycolysis enzymes: HK – hexokinase; PGI – glucose-6-phosphate isomerase; GPDH – glucose-6-phosphate degydrogenase; PFK – phosphofruktokinase; ALD – aldolase; TPI – triosephosphate isomerase; GAPDH – glyceraldehyde-3-phosphate dehydrogenase; DPGM – diphosphoglycerate mutase; PGK – phosphoglycerate kinase; DPGP – diphosphoglycerate phosphatase; PGM - phosphoglycerate mutase; ENO – enolase; PK – pyruvate kinase; LDH – lactate dehydrogenase. $V_{transpLAC}$ is a rate of lactate transport across the RBC membrane.

Table S2. Differential equations for the glycolysis metabolites in the presence of NADP-dependent glutamate dehydrogenase (NADP-GDH) and alanine aminotransferase (AAT), co-encapsulated into erythrocytes [a].

Variable	Differential equation
Pyruvate (PYR)	$\frac{d[PYR]}{dt} = V_{PK} - V_{LDH} + V_{AAT} - V_{transpPYR}$
Nicotinamide-adenine dinucleotide oxidised (NAD)	$\frac{d[NAD]}{dt} = V_{LDH} - V_{GAPDH}$
α-Ketoglutarate (AKG)	$\frac{d[AKG]}{dt} = V_{GDH} - V_{AAT} - V_{transpAKG}$
Glutamate (GLU)	$\frac{d[GLU]}{dt} = V_{AAT} - V_{NADP-GDH}$
Alanine (ALA)	$\frac{d[ALA]}{dt} = -V_{AAT} - V_{transpALA}$
Nicotinamide-adenine dinucleotide phosphate oxidised (NADP)	$\frac{d[NADP]}{dt} = V_{ox} - 2V_{GPDH} - V_{NADP-GDH}$
Ammonium (NH_3+NH_4^+) (AMM)	$\frac{d[AMM]}{dt} = V_{NaDP-GDH}$

[a] $V_{NADP\text{-}GDH}$ is a rate of NADP-dependent GDH. $V_{transpRYP}$, $V_{transpAKG}$, and $V_{transpALA}$ represent rates of PYR, AKG and ALA transport across the RBC membrane. V_{ox} is the oxidation rate for NADPH.

Table S3. Differential equations for the glycolysis metabolites in the presence of AAT and NAD-dependent GDH, co-encapsulated in erythrocytes [a)].

Variable	Differential equation
Nicotinamide-adenine dinucleotide oxidised (NAD)	$\frac{d[NAD]}{dt} = V_{LDH} - V_{GAPDH} - V_{NAD-GDH}$
Nicotinamide-adenine dinucleotide phosphate oxidised (NADP)	$\frac{d[NADP]}{dt} = V_{ox} - 2V_{GPDH}$

[a)] The equations for metabolites that are absent in Table 3 coincide with the corresponding equations in Table S2. $V_{NAD\text{-}GDH}$ is the rate for NAD-dependent GDH.

Table S4. Differential equations for the glycolysis metabolites in the presence of co-encapsulated in erythrocytes universal GDH and AAT [a)].

Variable	Differential equation
Pyruvate (PYR)	$\frac{d[PYR]}{dt} = V_{PK} - V_{LDH} + V_{AAT} - V_{transpPYR}$
Nicotinamide-adenine dinucleotide oxidised (NAD)	$\frac{d[NAD]}{dt} = V_{LDH} - V_{GAPDH} - V_{GDH-UNI}^{NAD}$
Nicotinamide-adenine dinucleotide phosphate oxidised (NADP)	$\frac{d[NADP]}{dt} = V_{ox} - 2V_{GPDH} - V_{GDH-UNI}^{NADP}$
Glutamate (GLU)	$\frac{d[GLU]}{dt} = V_{AAT} - V_{GDH-UNI}^{NAD} - V_{GDH-UNI}^{NADP}$
Alanine (ALA)	$\frac{d[ALA]}{dt} = -V_{AAT} - V_{transpALA}$
Adenosine triphosphate (ATP)**[)]	$\frac{d[ATP]}{dt} = V_{PGK} + V_{PK} - V_{HK} - V_{PFK} - V_{ATP} - V_{NaKATP}$
Ammonium (AMM)	$\frac{d[AMM]}{dt} = V_{GDH-UNI}^{NAD} + V_{GDH-UNI}^{NADP}$

[a)] Universal GDH (GDH-UNI) is simultaneously NAD- and NADP-dependent. $V_{GDH-UNI}^{NAD}$ and $V_{GDH-UNI}^{NADP}$ are shares of the rate of universal GDH, associated with the NAD- and NADP-components of the system.

**[)] The ADP and AMP concentrations were calculated assuming that a pool of adenylates was constant, and the equilibrium constant for adenylate kinase was: $K_{eq}=[ADP]^2/[AMP][ATP]=1$. V_{NaKATP} is the rate of Na^+-K^+-ATPase.

Table S5. Differential equations for the glycolysis metabolites in erythrocytes with encapsulated alanine dehydrogenase (ADH).

Variable	Differential equation
Pyruvate (PYR)	$\frac{d[PYR]}{dt} = V_{PK} - V_{LDH} + V_{ADH} - V_{transpPYR}$
Nicotinamide-adenine dinucleotide oxidised (NAD)	$\frac{d[NAD]}{dt} = V_{LDH} - V_{GAPDH} - V_{ADH}$
Adenosine triphosphate (ATP)	$\frac{d[ATP]}{dt} = V_{PGK} + V_{PK} - V_{HK} - V_{PFK} - V_{ATP} - V_{NaKATP}$
Ammonium (AMM)	$\frac{d[AMM]}{dt} = V_{ADH}$

Table S6. Differential equations for the glycolysis metabolites in erythrocytes with encapsulated glutamine synthetase (GS).

Variable	Differential equation
Glutamate (GLU)	$\frac{d[GLU]}{dt} = -V_{GS}$
Glutamine (GLN)	$\frac{d[GLN]}{dt} = V_{GS} - V_{transpGLN}$
Adenosine triphosphate (ATP)	$\frac{d[ATP]}{dt} = V_{PGK} + V_{PK} - V_{HK} - V_{PFK} - V_{ATP} - V_{NaKATP} - V_{GS}$
Ammonium (AMM)	$\frac{d[AMM]}{dt} = -V_{GS}$

Table S7. Equations for the enzymes of glycolysis, pentose phosphate pathway and the processes, that consume energy, in erythrocytes [a)].

Hexokinase

$$V_{HK} = a_{HK} \frac{[ATP]/K_{HK}^{1}}{1+[ATP]/K_{HK}^{1} + [G6P]/K_{HK}^{2}}$$

$a_{HK} = 12$ *mM/h*, $K_{HK}^{1} = 1$ *mM*, $K_{HK}^{2} = 5.5 \cdot 10^{-3}$ *mM*

Glucose-6-phosphate isomerase

$$V_{GPI} = a_{GPI} \frac{([G6P]-[F6P]K_{GPI}^{1})/K_{GPI}^{2}}{1+[G6P]/K_{GPI}^{2}+[F6P]/K_{GPI}^{3}}$$

$a_{GPI} = 360$ *mM/h*, $K_{GPI}^{1} = 3$ *mM*, $K_{GPI}^{2} = 0.3$ *mM*, $K_{GPI}^{3} = 0.2$ *mM*

Phosphofructokinase

$$V_{PFK} = a_{PFK} \frac{1.1*[ATP][F6P]\left(\frac{1}{1+[AMP]/K_{PFK}^{3}}+\frac{2[AMP]}{K_{PFK}^{3}+[AMP]}\right)}{(K_{PFK}^{2}+[ATP])(K_{PFK}^{1}+[F6P])\left(1+10^{8}\frac{(1+[ATP/K_{PFK}^{4}])^{4}}{(1+[AMP]/K_{PFK}^{3})^{4}(1+[F6P]/K_{PFK}^{5})^{4}}\right)}$$

$a_{PFK} = 380$ *mM/h*, $K_{PFK}^{1} = 0.1$ *mM*, $K_{PFK}^{2} = 2$ *mM*, $K_{PFK}^{3} = 0.01$ *mM*, $K_{PFK}^{4} = 0.195$ *mM*, $K_{PFK}^{5} = 3.7 \cdot 10^{-4}$ *mM*

Aldolase

$$V_{ALD} = a_{ALD} \frac{[FDP]/K_{ALD}^{1}-[DAP][GAP]/K_{ALD}^{2}}{1+\frac{[FDP]}{K_{ALD}^{3}}+\frac{[DAP]}{K_{ALD}^{4}}+\frac{[GAP]}{K_{ALD}^{5}}+\frac{[FDP][DAP]}{K_{ALD}^{3}K_{ALD}^{4}}+\frac{[DAP]^{2}}{K_{ALD}^{4}K_{ALD}^{6}}+\frac{[DAP][GAP]}{K_{ALD}^{4}K_{ALD}^{7}}}$$

$a_{ALD} = 76$ *mM/h*, $K_{ALD}^{1} = 2 \cdot 10^{-4}$ *mM*, $K_{ALD}^{2} = 1.2 \cdot 10^{-5}$ mM^{2}, $K_{ALD}^{3} = 0.01$ *mM*, $K_{ALD}^{4} = 0.032$ *mM*, $K_{ALD}^{5} = 2.1 \cdot 10^{-3}$ *mM*, $K_{ALD}^{6} = 2$ *mM*, $K_{ALD}^{7} = 0.065$ *mM*

Triosophosphateisomerase

$$V_{TPI} = a_{TPI} \frac{([DAP]-[GAP]/K_{TPI}^{2})/K_{TPI}^{1}}{1+[DAP]/K_{TPI}^{1}+[GAP]/K_{TPI}^{3}}$$

$a_{TPI} = 3000$ *mM/h*, $K_{TPI}^{1} = 0.82$ *mM*, $K_{TPI}^{2} = 0.45$ *mM*, $K_{TPI}^{3} = 0.43$ *mM*

Glyceraldehyde phosphate dehydrogenase

$$V_{GAPDH} = a_{GAPDH} \frac{([GAP][NAD][P_{i}]-[1,3DPG][NADH]/K_{GAPDH}^{4})/K_{GAPDH}^{1}K_{GAPDH}^{2}K_{GAPDH}^{3}}{1.29\left(1+\frac{[GAP]}{K_{GAPDH}^{1}}+\frac{[1,2DPG]}{K_{GAPDH}^{5}}\right)\left(1+\frac{[NAD]}{K_{GAPDH}^{2}}+\frac{[NADH]}{K_{GAPDH}^{6}}\right)}$$

$a_{GAPD} = 690$ *mM/h*, $K_{GAPD}^{1} = 0.13$ *mM*, $K_{GAPD}^{2} = 0.13$ *mM*, $K_{GAPD}^{3} = 3.4$ *mM*, $K_{GHAPD}^{4} = 0.136$ *mM*, $K_{GAPD}^{5} = 0.013$ *mM*, $K_{GAPD}^{6} = 2 \cdot 10^{-3}$

Phosphoglycerate kinase

$$V_{PGK} = a_{PGK} \frac{([1{,}3DPG][ADP] - [3PG][ATP]/K_{PGK}^{3})/K_{PGK}^{1}K_{PGK}^{2}}{1+[ATP]/K_{PGK}^{5} + [ADP]/K_{PGK}^{2} + A[1{,}3DPG]/K_{PGK}^{1} + B[3PG]/K_{PGK}^{6}}$$

$$A = (K_{PGK}^{4} + [ADP] + K_{PGK}^{4}[ATP]/K_{PGK}^{5})/K_{PGK}^{2},$$

$$B = (K_{PGK}^{7} + [ATP] + K_{PGK}^{7}[ADP]/K_{PGK}^{2})/K_{PGK}^{5}$$

$a_{PGK} = 7330$ *mM/h*, $K_{PGK}^{1} = 2.2 \cdot 10^{-3}$ mM, $K_{PGK}^{2} = 0.14$ mM, $K_{PGK}^{3} = 380$ mM, $K_{PGK}^{4} = 0.3$ mM, $K_{PGK}^{5} = 0.27$ mM, $K_{PGK}^{6} = 1.4$ mM, $K_{PGK}^{7} = 0.4$ mM

Diphosphoglycerate mutase

$$V_{DPGM} = a_{DPGM} \frac{[1{,}3DPG]}{K_{DPGM}^{1} + K_{DPGM}^{2}[1{,}3DPG] + [2{,}3DPG]}$$

$a_{DPGM} = 3892$ *mM/h*, $K_{DPGM}^{1} = 0.04$ mM, $K_{DPGM}^{2} = 0.013$ mM

Diphosphoglycerate phosphatase

$$V_{DPGP} = a \frac{[2{,}3DPG]}{[2{,}3DPG] + K_{DPGP}^{1}(1+[2PG]+[3PG]/K_{DPGP}^{2})}$$

$a_{DPGP} = 0.65$ *mM/h*, $K_{DPGP}^{1} = 0.02$ mM, $K_{DPGD}^{2} = 6 \cdot 10^{-3}$

Phosphoglycerate mutase

$$V_{PGM} = a_{PGM} \frac{([3PG] - [2PG]/K_{PGM}^{2})/K_{PGM}^{1}}{1+[3PG]/K_{PGM}^{1} + [2PG]/K_{PGM}^{3}}$$

$a_{PGM} = 1100$ *mM/h*, $K_{PGM}^{1} = 0.27$ mM, $K_{PGM}^{2} = 0.24$, $K_{PGM}^{3} = 0.02$ mM

Enolase

$$V_{ENO} = a_{ENO} \frac{([2PG] - [PEP]/K_{ENO}^{2})/K_{ENO}^{1}}{1+[2PG]/K_{ENO}^{1} + [PEP]/K_{ENO}^{3}}$$

$a_{ENO} = 83$ *mM/h*, $K_{ENO}^{1} = 0.056$ mM, $K_{ENO}^{2} = 6.7$, $K_{ENO}^{3} = 2 \cdot 10^{-3}$ mM

Pyruvate kinase

$$V_{PK} = a_{PK} \frac{[PEP][ADP]/K_{PK}^{1}K_{PK}^{2}}{1+[ATP]/K_{PK}^{3} + [ADP]/K_{PK}^{2} + [PEP]/K_{PK}^{1} + [PEP][ADP]/K_{PK}^{1}K_{PK}^{2}}$$

$a_{PK} = 120$ *mM/h*, $K_{PK}^{1} = 0.05$ mM, $K_{PK}^{2} = 0.43$ mM, $K_{PK}^{3} = 0.35$ mM

**Lactate dehydrogenase**<br><br>$$V_{LDH} = a_{LDH} \frac{([PYR][NADH] - [LAC][NAD]/K_{LDH}^3)/K_{LDH}^1 K_{LDH}^2}{1 + \frac{[PYR]}{K_{LDH}^1} + \frac{[NADH]K_{LDH}^4 + [PYR][NADH] + [LAC][NADH]K_{LDH}^4/K_{LDH}^5}{K_{LDH}^1 K_{LDH}^2} + \frac{C}{K_{LDH}^5 K_{LDH}^6}}$$<br>$$C = K_{LDH}^7[NAD] + K_{LDH}^6[LAC] + [NAD][LAC] + K_{LDH}^7[PYR][NAD]/K_{LDH}^1$$<br><br>$a_{LDH} = 550$ mM/h, $K_{LDH}^1 = 0.022$ mM, $K_{LDH}^2 = 7 \cdot 10^{-3}$ mM, $K_{LDH}^3 = 426$, $K_{LDH}^4 = 0.14$ mM, $K_{LDH}^5 = 380$ mM, $K_{LDH}^6 = 0.1$ mM, $K_{LDH}^7 = 170$ mM
**Glucose-6-phosphate dehydrogenase**<br><br>$$V_{GPDH} = a_{GPDH} \frac{\frac{[G6P]}{K_{GPDH}^1}}{1 + \frac{[G6P]}{K_{GPDH}^1}} \cdot \frac{\frac{[NADP]}{K_{GPDH}^2}}{1 + \frac{[NADP]}{K_{GPDH}^2} + \frac{[NADPH]}{K_{GPDH}^3}}$$<br><br>$A_{GPDH} = 25$ mM/h, $K_{GPDH}^1 = 0.04$ mM, $K_{GPDH}^2 = 4 \cdot 10^{-3}$ mM, $K_{GPDH}^3 = 0.02$ mM
**Na^+-K^+-ATPase**<br><br>$$V_{NaKATP} = a_{NaKATP}[Na^+][ATP]$$<br>$$a_{NaKATP} = 0.045 \ mM/h$$
**All other ATP-ases are presented by the following equation**<br>$$V_{ATP} = a_{ATP} \frac{[ATP]}{[ATP] + K_{ATP}}$$<br>$a_{ATP} = 1.6$ mM/h, $K_{ATP} = 1$ mM

a) Data on glycolysis and processes with energy consumption was taken from[1].

Table S8. Equations for the description of enzymes encapsulated in erythrocytes [a)]

NADP-dependent glutamate dehydrogenase[2-5]

$$V_{GDH} = ([NADP][GLU] - [AMM][AKG][NADPH]/K_{GDH}^{eq})/D_{GDH}$$

$$D_{GDH} = K_{GDH}^{2}K_{GDH}^{3} + K_{GDH}^{3}[NADP] + K_{GDH}^{1}[GLU] + [NADP][GLU] +$$

$$+\frac{K_{GDH}^{2}K_{GDH}^{3}K_{GDH}^{7}[AMM]}{K_{GDH}^{5}K_{GDH}^{8}} + \frac{K_{GDH}^{2}K_{GDH}^{3}[NADPH]}{K_{GDH}^{10}} + \frac{K_{GDH}^{3}[NADP][AMM]}{K_{GDH}^{6}} +$$

$$+\frac{K_{GDH}^{2}K_{GDH}^{3}K_{GDH}^{9}[AMM][AKG]}{K_{GDH}^{5}K_{GDH}^{8}K_{GDH}^{10}} + \frac{K_{GDH}^{1}[GLU][NADPH]}{K_{GDH}^{10}} + \frac{K_{GDH}^{2}K_{GDH}^{3}[AKG][NADPH]}{K_{GDH}^{8}K_{GDH}^{10}} +$$

$$+\frac{K_{GDH}^{2}K_{GDH}^{3}K_{GDH}^{7}[AMM][NADPH]}{K_{GDH}^{5}K_{GDH}^{8}K_{GDH}^{10}} + \frac{[NADP][GLU][AMM]}{K_{GDH}^{8}} + \frac{K_{GDH}^{2}K_{GDH}^{3}[AMM][AKG][NADPH]}{K_{GDH}^{5}K_{GDH}^{8}K_{GDH}^{10}} +$$

$$+\frac{K_{GDH}^{3}K_{GDH}^{9}[NADP][AMM][AKG]}{K_{GDH}^{5}K_{GDH}^{8}K_{GDH}^{10}} + \frac{[NADP][GLU][AKG]}{K_{GDH}^{8}} + \frac{K_{GDH}^{2}K_{GDH}^{3}[GLU][AKG][NADPH]}{K_{GDH}^{4}K_{GDH}^{8}K_{GDH}^{10}} +$$

$$+\frac{[NADP][GLU][AMM][AKG]}{K_{GDH}^{6}K_{GDH}^{8}} + \frac{K_{GDH}^{2}K_{GDH}^{8}[GLU][AMM][AKG][NADPH]}{K_{GDH}^{4}K_{GDH}^{5}K_{GDH}^{8}K_{GDH}^{10}}$$

$K_{GDH}^{eq} = 4\cdot 10^{-3}\ mM$, $K_{GDH}^{1} = 0.028\ mM$, $K_{GDH}^{2} = 0.24\ mM$, $K_{GDH}^{3} = 0.74\ mM$, $K_{GDH}^{4} = 6.3\ mM$, $K_{GDH}^{5} = 6.5\ mM$, $K_{GDH}^{6} = 2.9\ mM$, $K_{GDH}^{7} = 2.4\ mM$, $K_{GDH}^{8} = 0.315\ mM$, $K_{GDH}^{9} = 0.022\ mM$, $K_{GDH}^{10} = 0.028\ mM$.

NAD/NADP-dependent glutamate dehydrogenase (item for NADP) [a)]

$$V_{GDH-UNI}^{NADP} = ([NADP][GLU] - [AMM][AKG][NADPH]/K_{GDH}^{eq})/D_{GDH}$$

$$D_{GDH} = K_{GDH}^{2}K_{GDH}^{3} + K_{GDH}^{3}\left([NADP]+[NAD]\right) + K_{GDH}^{1}[GLU] + \left([NADP]+[NAD]\right)[GLU] +$$

$$+\frac{K_{GDH}^{2}K_{GDH}^{3}K_{GDH}^{7}[AMM]}{K_{GDH}^{5}K_{GDH}^{8}} + \frac{K_{GDH}^{2}K_{GDH}^{3}\left([NADPH]+[NADH]\right)}{K_{GDH}^{10}} +$$

$$+\frac{K_{GDH}^{3}\left([NADP]+[NAD]\right)[AMM]}{K_{GDH}^{6}} + \frac{K_{GDH}^{2}K_{GDH}^{3}K_{GDH}^{9}[AMM][AKG]}{K_{GDH}^{5}K_{GDH}^{8}K_{GDH}^{10}} +$$

$$+\frac{K_{GDH}^{1}[GLU]\left([NADPH]+[NADH]\right)}{K_{GDH}^{10}} + \frac{K_{GDH}^{2}K_{GDH}^{3}[AKG]\left([NADPH]+[NADH]\right)}{K_{GDH}^{8}K_{GDH}^{10}} +$$

$$+\frac{K_{GDH}^{2}K_{GDH}^{3}K_{GDH}^{7}[AMM]\left([NADPH]+[NADH]\right)}{K_{GDH}^{5}K_{GDH}^{8}K_{GDH}^{10}} + \frac{\left([NADP]+[NAD]\right)[GLU][AMM]}{K_{GDH}^{8}} +$$

$$+\frac{K_{GDH}^{2}K_{GDH}^{3}[AMM][AKG]\left([NADPH]+[NADH]\right)}{K_{GDH}^{5}K_{GDH}^{8}K_{GDH}^{10}} + \frac{K_{GDH}^{3}K_{GDH}^{9}\left([NADP]+[NAD]\right)[AMM][AKG]}{K_{GDH}^{5}K_{GDH}^{8}K_{GDH}^{10}} +$$

$$+\frac{\left([NADPH]+[NADH]\right)[GLU][AKG]}{K_{GDH}^{8}} + \frac{K_{GDH}^{2}K_{GDH}^{3}[GLU][AKG]\left([NADPH]+[NADH]\right)}{K_{GDH}^{4}K_{GDH}^{8}K_{GDH}^{10}} +$$

$$+\frac{[\left([NADP]+[NAD]\right)[GLU][AMM][AKG]}{K_{GDH}^{6}K_{GDH}^{8}} + \frac{K_{GDH}^{2}K_{GDH}^{8}[GLU][AMM][AKG]\left([NADPH]+[NADH]\right)}{K_{GDH}^{4}K_{GDH}^{5}K_{GDH}^{8}K_{GDH}^{10}}$$

Alanine aminotransferase[6]

$$V_{AAT} = v_{AAT}^F v_{AAT}^R ([ALA][AKG] - [PYR][GLU]/K_{AAT}^{eq})/D_{AAT}$$

$$D_{AAT} = v_{AAT}^R K_{AAT}^1 [AKG] + v_{AAT}^R K_{AAT}^2 [ALA] + v_{AAT}^R [AKG][ALA] +$$
$$+ \frac{v_{AAT}^F K_{AAT}^5 [GLU]}{K_{AAT}^{eq}} + \frac{v_{AAT}^F K_{AAT}^3 [PYR]}{K_{AAT}^{eq}} + \frac{v_{AAT}^F [PYR][GLU]}{K_{AAT}^{eq}} +$$
$$+ \frac{v_{AAT}^R K_{AAT}^1 [AKG][GLU]}{K_{AAT}^4} + \frac{v_{AAT}^R K_{AAT}^2 [ALA][PYR]}{K_{AAT}^6} + \frac{v_{AAT}^R K_{AAT}^2 [ALA]^2}{K_{AAT}^7} +$$
$$+ \frac{v_{AAT}^F K_{AAT}^5 [GLU]^2}{K_{AAT}^{eq} K_{AAT}^8} + \frac{v_{AAT}^R K_{AAT}^2 [ALA][GLU]}{K_{AAT}^9}$$

$v_{AAT}^F = 2.46 \cdot 10^{-3}$ *mM/h*, $v_{AAT}^R = 2.1 \cdot 10^{-3}$ *mM/h*, $K_{AAT}^1 = 10$ *mM*, $K_{AAT}^2 = 0.12$ *mM*, $K_{AAT}^3 = 8.1$ *mM*, $K_{AAT}^4 = 2.8$ *mM*, $K_{AAT}^5 = 0.23$ *mM*, $K_{AAT}^6 = 0.23$ *mM*, $K_{AAT}^7 = 470$ *mM*, $K_{AAT}^8 = 96$ *mM*, $K_{AAT}^9 = 57$ *mM* $K_{AAT}^{eq} = 2.2$

Alanine dehydrogenase[7]

$$V_{ADH} = a_{ADH} \frac{v_{ADH}^F [NAD][ALA]/K_{ADH}^2 / K_{ADH}^3 - v_{ADH}^R [AMM][PYR][NADH]/K_{ADH}^5 / K_{ADH}^8 / K_{ADH}^{10}}{D_{ADH}}$$

$$D_{ADH} = 1 + \frac{[NAD]}{K_{ADH}^2} + \frac{K_{ADH}^1 [ALA]}{K_{ADH}^2 K_{ADH}^3} + \frac{K_{ADH}^7 [AMM]}{K_{ADH}^5 K_{ADH}^8} + \frac{NADH}{K_{ADH}^{10}} + \frac{K_{ADH}^7 [NAD][AMM]}{K_{ADH}^2 K_{ADH}^5 K_{ADH}^8} +$$
$$+ \frac{K_{ADH}^1 [ALA][NADH]}{K_{ADH}^2 K_{ADH}^3 K_{ADH}^{10}} + \frac{K_{ADH}^9 [AMM][PYR]}{K_{ADH}^5 K_{ADH}^8 K_{ADH}^{10}} + \frac{K_{ADH}^7 [AMM][NADH]}{K_{ADH}^5 K_{ADH}^8 K_{ADH}^{10}} + \frac{[PYR][NADH]}{K_{ADH}^8 K_{ADH}^{10}} +$$
$$+ \frac{K_{ADH}^7 [NAD][ALA][AMM]}{K_{ADH}^2 K_{ADH}^4 K_{ADH}^5 K_{ADH}^8} + \frac{K_{ADH}^6 K_{ADH}^9 [NAD][ALA][PYR]}{K_{ADH}^2 K_{ADH}^4 K_{ADH}^5 K_{ADH}^8 K_{ADH}^{10}} + \frac{K_{ADH}^9 [NAD][AMM][PYR]}{K_{ADH}^2 K_{ADH}^5 K_{ADH}^8 K_{ADH}^{10}} +$$
$$+ \frac{[ALA][PYR][NADH]}{K_{ADH}^2 K_{ADH}^8 K_{ADH}^{10}} + \frac{K_{ADH}^9 [NAD][ALA][AMM][PYR]}{K_{ADH}^2 K_{ADH}^4 K_{ADH}^5 K_{ADH}^8 K_{ADH}^{10}} + \frac{K_{ADH}^1 [ALA][AMM][PYR][NADH]}{K_{ADH}^2 K_{ADH}^3 K_{ADH}^6 K_{ADH}^8 K_{ADH}^{10}} +$$
$$+ \frac{[NAD][ALA]}{K_{ADH}^2 K_{ADH}^3} + \frac{[AMM][PYR][NADH]}{K_{ADH}^5 K_{ADH}^8 K_{ADH}^{10}}$$

$v_{ADH}^F = 1.728 \cdot 10^5$ h^{-1}, $v_{ADH}^R = 3.6 \cdot 10^6$ h^{-1}, $K_{ADH}^1 = 0.22$ *mM*, $K_{ADH}^2 = 0.33$ *mM*, $K_{ADH}^3 = 4.4$ *mM*, $K_{ADH}^4 = 75$ *mM*, $K_{ADH}^5 = 160$ *mM*, $K_{ADH}^6 = 160$ *mM*, $K_{ADH}^7 = 0.44$ *mM*, $K_{ADH}^8 = 0.3$ *mM*, $K_{ADH}^9 = 38$ *mM*, $K_{ADH}^{10} = 2.7$ *mM*.

Glutamine synthetase[8,9]

$$V_{GS} = a_{GS} \frac{v_{GS}^F [ATP][GLU][AMM]/K_{GS}^2 / K_{GS}^4 / K_{GS}^5 - v_{GS}^R [P][GLN][ADP]/K_{GS}^7 / K_{GS}^{10} / K_{GS}^{12}}{D_{GS}}$$

$$D_{GS}=1+\frac{[ATP]}{K_{GS}^{2}}+\frac{K_{GS}^{3}[GLU]}{K_{GS}^{4}}+\frac{K_{GS}^{9}[P]}{K_{GS}^{7}K_{GS}^{10}}+\frac{[ADP]}{K_{GS}^{11}}+\frac{[ATP][GLU]}{K_{GS}^{2}K_{GS}^{4}}+\frac{K_{GS}^{3}[ATP][AMM]}{K_{GS}^{2}K_{GS}^{4}K_{GS}^{5}}+$$
$$+\frac{K_{GS}^{9}[ATP][P]}{K_{GS}^{2}K_{GS}^{7}K_{GS}^{10}}+\frac{K_{GS}^{1}[GLU][AMM]}{K_{GS}^{2}K_{GS}^{4}K_{GS}^{5}}+\frac{K_{GS}^{3}[AMM][ADP]}{K_{GS}^{4}K_{GS}^{5}K_{GS}^{12}}+\frac{K_{GS}^{11}[P][GLN]}{K_{GS}^{7}K_{GS}^{10}K_{GS}^{12}}+\frac{K_{GS}^{9}[P][ADP]}{K_{GS}^{7}K_{GS}^{10}K_{GS}^{12}}+$$
$$+\frac{[GLN][ADP]}{K_{GS}^{10}K_{GS}^{12}}+\frac{K_{GS}^{9}[ATP][GLU][P]}{K_{GS}^{2}K_{GS}^{4}K_{GS}^{7}K_{GS}^{10}}+\frac{K_{GS}^{11}[ATP][P][GLN]}{K_{GS}^{2}K_{GS}^{7}K_{GS}^{10}K_{GS}^{12}}+\frac{K_{GS}^{1}[GLU][AMM][ADP]}{K_{GS}^{2}K_{GS}^{4}K_{GS}^{5}K_{GS}^{12}}+$$
$$+\frac{K_{GS}^{3}[AMM][GLN][ADP]}{K_{GS}^{4}K_{GS}^{5}K_{GS}^{10}K_{GS}^{12}}+\frac{K_{GS}^{9}[ATP][GLU][AMM][P]}{K_{GS}^{2}K_{GS}^{4}K_{GS}^{6}K_{GS}^{7}K_{GS}^{10}}+\frac{K_{GS}^{8}K_{GS}^{11}[ATP][GLU][AMM][GLN]}{K_{GS}^{2}K_{GS}^{4}K_{GS}^{6}K_{GS}^{7}K_{GS}^{10}K_{GS}^{12}}+$$
$$+\frac{K_{GS}^{11}[ATP][GLU][P][GLN]}{K_{GS}^{2}K_{GS}^{4}K_{GS}^{7}K_{GS}^{10}K_{GS}^{12}}+\frac{K_{GS}^{1}[GLU][AMM][GLN][ADP]}{K_{GS}^{2}K_{GS}^{4}K_{GS}^{5}K_{GS}^{10}K_{GS}^{12}}+$$
$$+\frac{K_{GS}^{1}[GLU][P][GLN][ADP]}{K_{GS}^{2}K_{GS}^{4}K_{GS}^{8}K_{GS}^{10}K_{GS}^{12}}+\frac{K_{GS}^{3}[AMM][P][GLN][ADP]}{K_{GS}^{4}K_{GS}^{5}K_{GS}^{8}K_{GS}^{10}K_{GS}^{12}}+$$
$$+\frac{K_{GS}^{11}[ATP][GLU][AMM][P][GLN]}{K_{GS}^{2}K_{GS}^{4}K_{GS}^{6}K_{GS}^{7}K_{GS}^{10}K_{GS}^{12}}+\frac{K_{GS}^{1}[GLU][AMM][P][GLN][ADP]}{K_{GS}^{2}K_{GS}^{4}K_{GS}^{5}K_{GS}^{8}K_{GS}^{10}K_{GS}^{12}}+$$
$$+\frac{[ATP][GLU][AMM]}{K_{GS}^{2}K_{GS}^{4}K_{GS}^{5}}+\frac{[P][GLN][ADP]}{K_{GS}^{7}K_{GS}^{10}K_{GS}^{12}}$$

$v_{GS}^{F}=5.2\cdot10^{4}\ h^{-1}$, $v_{GS}^{R}=5.94\cdot10^{3}\ h^{-1}$, $K_{GS}^{1}=0.079\ mM$, $K_{GS}^{2}=0.07\ mM$, $K_{GS}^{3}=3.1\ mM$, $K_{GS}^{4}=2.7\ mM$, $K_{GS}^{5}=0.22\ mM$, $K_{GS}^{6}=?$, $K_{GS}^{7}=3\ mM$, $K_{GS}^{8}=45\ mM$, $K_{GS}^{9}=2.5\ mM$, $K_{GS}^{10}=20\ mM$, $K_{GS}^{11}=0.044\ mM$, $K_{GS}^{12}=0.12\ mM$.

a) The rate for universal NAD- and NADP-dependent glutamate dehydrogenase ($V_{GDH-UNI}$) may be presented by the equation: $V_{GDH-UNI}=V_{GDH-UNI}^{NADP}+V_{GDH-UNI}^{NAD}$, where $V_{GDH-UNI}^{NAD}$ and $V_{GDH-UNI}^{NADP}$ are the rates connected with NAD- or NADP-dependent activities of this enzyme, respectively. The equation for $V_{GDH-UNI}^{NAD}$ may be obtained from corresponding equation for $V_{GDH-UNI}^{NADP}$ using the following replacements: $NAD\leftrightarrow NADP$, $NADH\leftrightarrow NADPH$.

Determination of the steady-state rate of ammonium processing

The stationary rate of ammonium processing (V_{AMM}) can be achieved only under conditions of a quasi-stationary state *in vivo*, if the ammonium concentration is constant (in other words, if the system constantly has an ammonium inflow). This rate is determined, as shown in Fig. S1.

Dependence of the stationary V_{AMM} on the relative AKG-permeability of the RBC membrane

The stationary V_{AMM} for erythrocyte bioreactors (EBRs), loaded with GDH and AAT, is limited by the permeability of the RBC membrane for AKG. The entire calculated curve is

shown in Fig. S2. The inset shows a part of this curve with the linear dependence of the V_{AMM} on AKG-permeability.

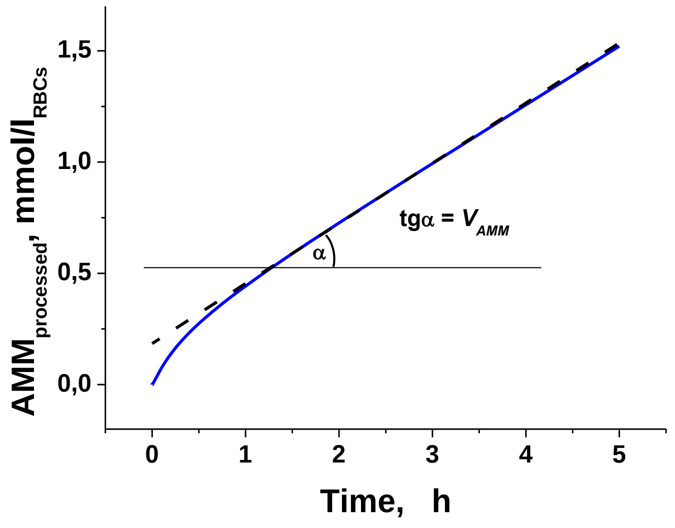

Fig. S1. Determination of the stationary rate of ammonium processing (V_{AMM}). The stationary rate was determined as slope of stationary part of the curve of an increase in the concentration of processed ammonium ($AMM_{processed}$) over time.

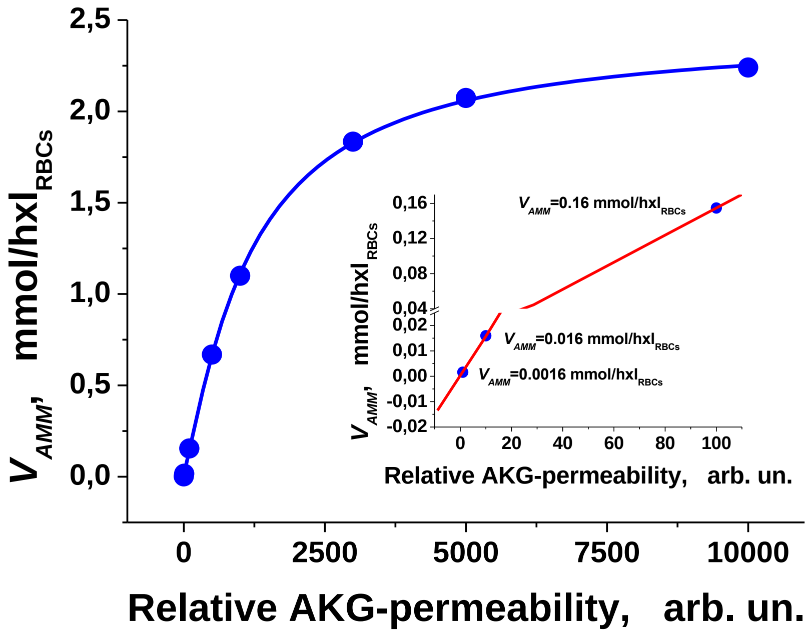

Fig. S2. The stationary rate of ammonium processing in the presence of EBRs containing NADP-dependent GDH depends on the relative RBC membrane permeability for AKG. The permeability of the erythrocyte membrane for AKG in normal physiological conditions was accepted as the unit permeability.

In practice, we can not significantly change the membrane permeability, however, if this could be done, this could not correct the situation, because even the 100-fold increase in membrane permeability leads to a very low rate of ammonium removal. For maximum increasing in V_{AMM} (to ~2.25 mmol/h×l_{RBCs}), the membrane permeability for AKG should be increased by 10^4 times, that is hardly achievable (Fig. S2).

Accumulation of glutamate into EBRs with GDH

With an increase in the permeability of the erythrocyte membrane for AKG, the rate of GLU accumulation within the erythrocyte containing GDH will increase (Fig. S3). Accumulation of this metabolite inside the cell will lead to a shift in the equilibrium of the reaction toward ammonium formation. In physiological conditions the erythrocyte has an excess of surface area relative to its volume (in 1.8-2 times). Thus, RBC may be osmotically destroyed if relative cell

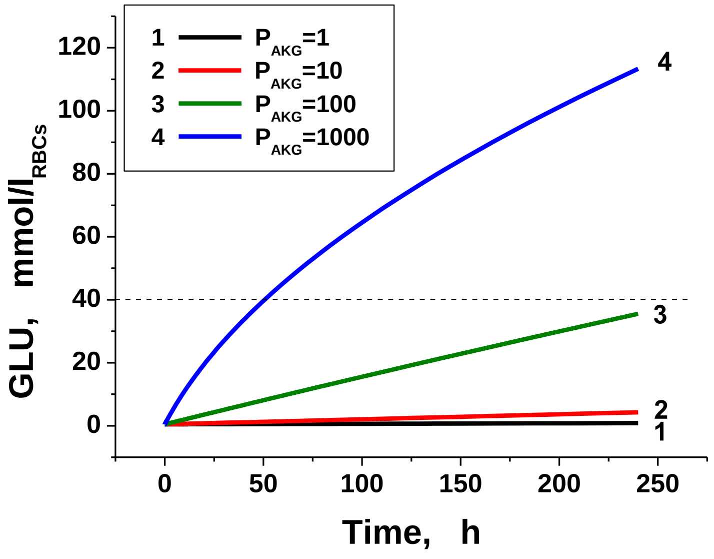

Fig. S3. The accumulation of GLU inside ammocytes containing GDH at different relative RBC membrane permeability for AKG. The GDH activity is 10 IU/ml_{RBCs}. The relative RBC membrane permeability for AKG (P_{AKG}) was equal: 1 – to the normal physiological permeability (P_{AKG}=1 arb. un.); 2 - P_{AKG}=10 arb. un.; 3 – P_{AKG}=100 arb. un.; 4 – P_{AKG}=1000 arb. un.

volume increases more than 1.8-2 times. Model calculations of increasing RBC volume with an increase in the concentration of non-penetrating molecules of different charge inside the cell are presented in Fig. S4. They show, that theoretically, the accumulation of GLU inside erythrocytes in concentrations above 40-50 mmol/l_{RBCs} can cause accelerated cell destruction due to impaired osmotic balance between cells and the external environment[1] (Fig. S4).

However, this is hardly possible in real conditions. With the physiological permeability of the membrane for AKG, accumulating such concentration of GLU within the erythrocyte will take a very long time (about several months). Theoretically, the time to reach this concentration decreases with increasing permeability of the membrane for AKG by 100 or more times. The intracellular concentration of GLU 40 mmol/l_{RBCs} can be achieved for ~270 or 50 h at a 100- and 1000-fold increase in this permeability, respectively (Fig. S3).

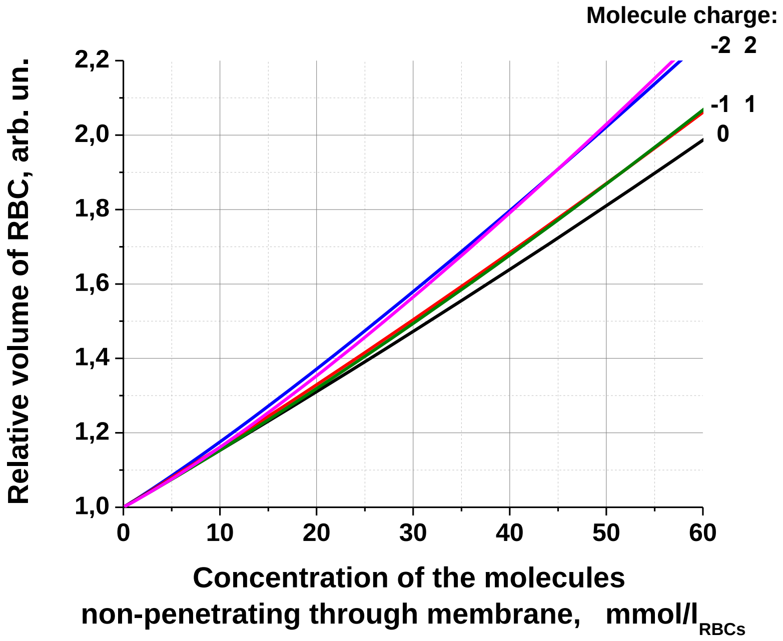

Fig. S4. An increase in the relative erythrocyte volume with an increase in the concentration of non-penetrating molecules of different charge accumulated into RBC.

Erythrocytes and bioreactors quality evaluation

Quality was studied for the initial erythrocytes, bioreactors loaded with GDH and AAT, as well as control erythrocytes, which underwent a complete protein encapsulation procedure, but in the presence of 1.1 mM albumin instead of GDH and AAT. Haematological indices and osmotic resistance of the cells, as well as the activity of the included enzymes in the cells were considered to be quality indicators. These parameters were evaluated not only for the initial RBCs, or fresh ammocytes, but also during the storage of suspensions of native and control RBCs and EBRs. Each of the samples was resuspended to a final haematocrit of 10% in a solution containing 137 mM NaCl, 2.7 mM KCl, 10 mM Na_2HPO_4, 2 mM KH_2PO_4, 1.3 mM $CaCl_2$, 5 mM $MgCl_2$, 10 mM glucose, 5% (w/w) BSA, 30 mM HEPES, 0.28 mM adenine and 0.02 mg/ml ampicillin (pH 7.4) and stored at 4°C for 6-7 days. On different days of storage,

osmotic fragility, haematological indices and intracellular activity of GDH and AAT were measured. The results are shown in Figs S5 and S6.

Preservation of GDH and AAT activity inside bioreactors at storage

The activity of each enzyme in EBRs was measured spectrophotometrically as described in the main text (Methods), on the day of preparation and during the weekly storage (see above). The results obtained showed that intracellular activity decreased relatively quickly in the first two days of storage. Then this decrease almost ceased. After 6 days of storage (i.e., on the seventh day after EBRs preparation), the activity of AAT and GDH decreased on average by about 24 and 50%, respectively (Fig. S5a).

Haematological indices

Haematological indices of the initial and control RBCs, as well as EBRs, were measured using the automatic haematological analyser Micros OT (ABX-France, Montpellier, France). The mean cell volume (MCV in fl) and the mean cell haemoglobin concentration (MCHC, in g/dl) in EBRs and control erythrocytes were about 30% lower than those in native RBCs. Especially strong (~ by 45% compared to native RBCs) was a decrease in the mean cellular haemoglobin (MCH, in pg) for cells treated with hypoosmotic dialysis (Figs S5b, S5d, and S5c, respectively). Included enzymes did not affect haematological indices, since the values of these indices in EBRs were very close to those in control RBCs that did not contain GDH and AAT.

The decrease in indices of the EBRs was slightly higher than previously reported when GDH was included in mouse erythrocytes[10]. This may indicate that the dialysis procedure in our case was slightly more stringent, because was performed at a lower osmolality of the dialysis solution (65 mOsm/kg compared to ~100 mOsm/kg in the study[10]). Despite the fact that during the enzyme encapsulation the RBCs indices were changed sufficiently, further decrease of these indices virtually did not occur during the storage of EBRs and control RBCs at 4°C (Figs S5b-d).

Osmotic fragility curves of the initial RBCs and EBRs with GDH and AAT

The osmotic fragility curves of the original (native) erythrocytes and EBRs obtained were measured as dependence of the value of lysed cells fraction on the osmolality of the solution. These fractions were measured after the addition of the cell suspension to a series of saline solutions with different osmolality. The measurements were performed on an Biochrom® Anthos Zenyth 340rt microplate reader (Biochrom, Ltd., Cambridge, UK).

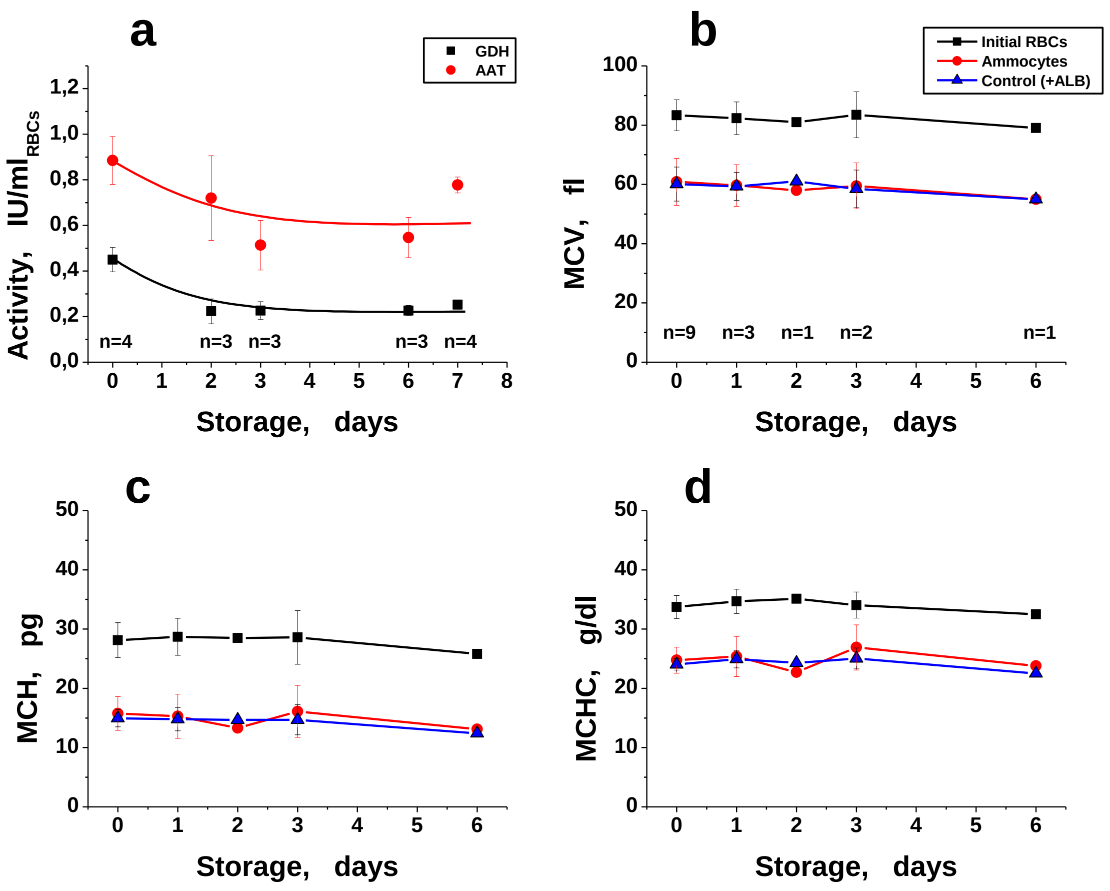

Fig. S5. Changes in the parameters of the initial erythrocytes, EBRs and control erythrocytes when stored in suspensions with haematocrit 10% at 4°C. Like EBRs, control RBCs underwent an encapsulation procedure, but in the presence of 1.1 mM albumin instead of GDH and AAT. EBRs and control erythrocytes were prepared on day 0. The activity of GDH and AAT in the initial suspension before dialysis was 10 $IU/ml_{suspension}$ and 5 $IU/ml_{suspension}$, respectively. (**a**) - Changes in the activity of GDH and AAT in the EBRs during storage. The mean values±SEM are presented. (**b**, **c**, and **d**) - Changes in erythrocyte indices (mean cell volume (MCV, in fl), mean haemoglobin content in the cell (MCH in pg) and mean haemoglobin concentration in the cell (MCHC, in g/dl), respectively) during storage of the cell suspensions of the initial and control erythrocytes, as well as EBRs. The distributions of all the measured parameters were normal according to the D'Agostino-Pearson test (MedCalc Statistical Software bvba, version 14.12, Belgium). The mean±SD is presented. The number of corresponding experiments is shown in panels **a,** and **b**.

To a number of NaCl solutions of different osmolality (from 0 to 300 mOsm/kg) (980 µl each), a cell suspension with a haematocrit of 5% was added (on 20 µl). The resulting suspensions were incubated for 30 min at room temperature. Then the samples were centrifuged (8 min at 1000 g) and absorbance was measured in supernatants at λ=540 nm (D_{540}). The

percentage of lysed cells was determined as the ratio (in %) of D_{540} measured in the supernatant of a specific sample to D_{540} of the same sample at 100% lysis (in a solution with zero osmolality). The value of osmotic resistance was characterised by the osmolality of the solution, at which 50% of the original cells were lysed (Hc_{50}). The osmolality of solutions was determined using a Vapor Pressure Osmometer (Vapro 5600) (Wescor Inc., Logan, UT, USA).

The osmotic fragility curves (Fig. S6a) were measured on different days of storage. In order not to overload the figure, only the averaged curves for day 0 (day of the EBRs preparation) and the curves obtained after 6 days of storage are given. The shape of the osmotic resistance curve for the EBRs on the day of their production differs significantly from the curve for the initial native RBCs. However, during storage, the curve for EBRs changes its shape and shifts to the left (Fig. S6a). After 6 days of storage, these curves hardly differ at the 50% level of haemolysis, but the EBRs have a slightly higher resistance to low osmolality than the suspension of the initial RBCs. The osmolality values, at which 50% haemolysis occurs, were somewhat decreased for both curves at storage, but this decrease was not very significant (Fig. S6b).

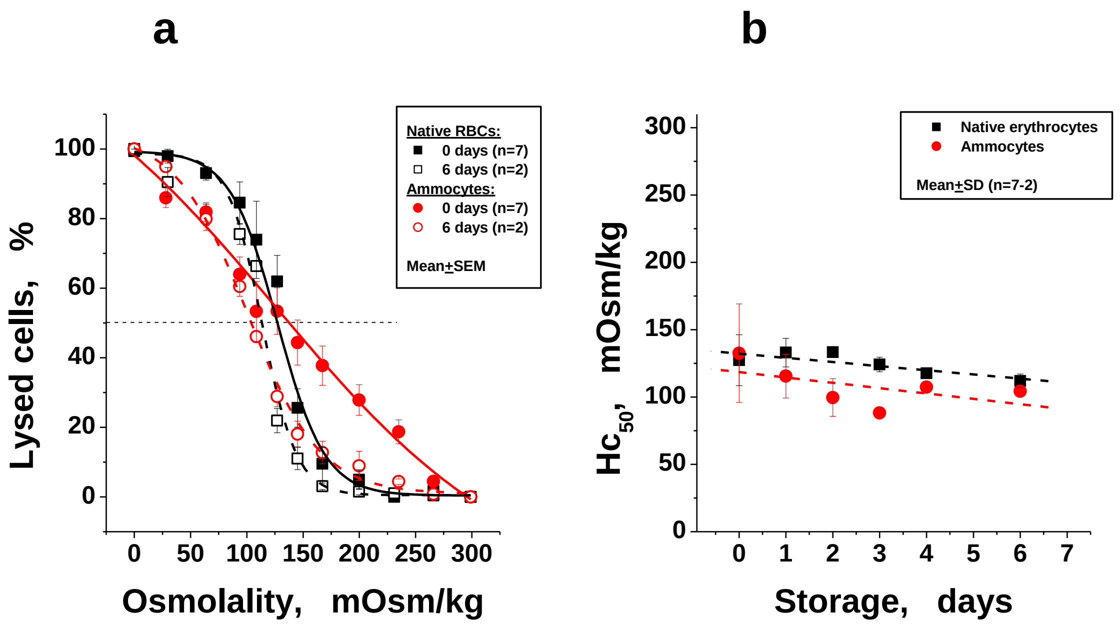

Fig. S6. The osmotic fragility of native RBCs and EBRs during the storage. (**a**) – The osmotic fragility curves of native RBCs and EBRs with GDH and AAT on the day of the procedure (day 0), and after 6 days of the storage of suspensions with haematocrit 10% at 4°C. EBRs were obtained by hypoosmotic dialysis (the enzyme concentration in the initial suspension was 10 $IU/ml_{suspension}$ and 5 $IU/ml_{suspension}$, for GDH and AAT, respectively). The mean values±SEM are presented. (**b**) – Changes during the storage in the values of osmolality, which causes 50% haemolysis of cells (Hc50) (mean±SD).

The activity of some glycolytic enzymes and the concentrations of metabolites used in the mouse erythrocyte-bioreactor model

Table S9. The activity of some glycolysis enzymes in human and mouse erythrocytes

N	Enzyme	V_{max}, nM/h×l_{RNCs}	
		Human [1]	Mouse [11]
1	Hexokinase	12	66
2	Glucose phosphate isomerase	360	1872
3	Phosphofructokinase	380	127
4	Aldolase	76	63
5	Triosephosphate isomerase	3000	37200
6	Glyceraldehyde-3-phosphate dehydrogenase	690	3534
7	Phosphoglycerate kinase	7330	4518
8	Phosphoglycerate mutase	1100	176
9	Enolase	83	642
10	Pyruvate kinase	120	2460
11	Lactate dehydrogenase	550	6720
12	Glucose-6-phosphate dehydrogenase	25	443

Table S10. Concentrations of some metabolites of glycolysis in human and mouse erythrocytes

N	Metabolite	Concentration into erythrocytes, mmol/l_{RBCs}	
		Human*⁾	Mouse [12, 13, 14]
1	G6P	0.02-0.11	0.079 [12]
2	F6P	0.006-0.016	0.046 [12]
3	FDP	0.002-0.03	0.017 [12]
4	DAP	0.0076-0.035	0.034 [12]
5	GAP	0.0048-0.02	0.015 [12]
6	2,3-DPG	4.17-5.7	9.4 [12]
7	3-PG	0.055-0.069	0.047 [12]
8	2-PG	0.0055-0.012	0.017 [12]
9	PEP	0.012-0.018	0.022 [12]
10	PYR	0.047-0.950	0.1 [12]
11	LAC	1.00-2.14	4.3 [12]
12	ATP	1.07-1.8	1.4 [12], 1.7 [13]
13	ADP	0.085-0.3	0.2 [12], 0.15 [13]
14	AMP	0.01-0.05	0.026 [13]
15	NADP + NADPH	0.0353-0.073	0.036 [13]
16	NAD + NADH	0.033-0.146	0.25 [12], 0.38 [13]
17	ALA	0.275-0.435	0.2 [14]
18	GLU	0.212-0.463	0.1 [14]

*⁾ All parameter values are presented in Table 1 and Table 2 in the main text.

Table S11. Medications currently used to reduce ammonium in the patients' blood.

	Preparation	Dose	Decrease of study-state ammonium concentration	Reference
1	Lactulose	30-60 ml<br>3 times/day	Initial arterial ammonia<br>115.7±22.7 µM<br>After 10 day of treatment<br>105.8±35.3 µM.<br>Decrease – 10 µM/10 days	Sharma B.C., et al. A randomized, double-blind, controlled trial comparing rifaximin plus lactulose with lactulose alone in treatment of overt hepatic encephalopathy. *Am. J. Gastroenterol*. **108,** 1458-1463 (2013).
2	Lactitol	20 g<br>3 times/day<br>(5-10 days)	Initial ammonia<br>124.1 (35-661.5) µg/dl<br>After minimum 5 day treatment<br>109 (12-432.7) µg/dl.<br>Decrease –15.1 µg/dl/5 days<br>(or 8.9 µM/5 days)	Mas A., et al. Comparison of rifaximin and lactitol in the treatment of acute hepatic encephalopathy: results of a randomized, double-blind, double-dummy, controlled clinical trial. *J. Hepatol.* **38,** 51-58 (2003).
3	Lactulose + rifaximin	Lactulose:<br>30-60 ml<br>3 times/day<br>+<br>rifaximin:<br>1200 mg/day	Initial arterial ammonia<br>132.6±29.8 µM<br>After 10 day of treatment<br>114.8±54.4 µM<br>Decrease – 18 µM/10 days	Sharma, B. C., et al. A randomized, double-blind, controlled trial comparing rifaximin plus lactulose with lactulose alone in treatment of overt hepatic encephalopathy. *Am. J. Gastroenterol*. **108,** 1458-1463 (2013).
4	Rifaximin	400 mg<br>3 times/day<br>(5-10 days)	Initial ammonia was<br>120.5 (12.1-300) µg/dl.<br>After minimum 5 day of treatment - 69.5 (13-268).<br>Decrease – 51 µg/dl/5 days<br>(or 30.0 µM/5 days)	Mas, A., et al. Comparison of rifaximin and lactitol in the treatment of acute hepatic encephalopathy: results of a randomized, double-blind, double-dummy, controlled clinical trial. J. *Hepatol.* **38,** 51-58 (2003).
5	Neomycin<br><br>or<br>Erythromy-cin	1 g<br>4 times/day<br><br>or<br>250 mg<br>4 times/day	Decrease in serum:<br>30.70±92.15 µM<br>during 6-7 days<br><br>31.4±49.32 µM<br>during 6-7 days.	Romeiro, F. G., et al. Erythromycin versus neomycin in the treatment of hepatic encephalopathy in cirrhosis: A randomized double-blind study. *BMC gastroenterology*. **13,** 13 (2013 Jan 16). doi: 10.1186/1471-230X-13-13.
6	Mannite solution	2000 ml/day<br>(1-2 days)	With mannite ammonium decreased from 156.4±98.0 µg/dl to 110.0±24.2 µg/dl (46.4 µg/dl/day or 27.3 µM/day).	Tromm, A., et al. Orthograde whole gut irrigation with mannite versus paromomycine + lactulose as prophylaxis of hepatic encephalopathy in patients with cirrhosis and upper gastrointestinal bleeding: results

	or paromomy-cin + lactulose	1 g<br>3 times/day,<br>10 ml<br>3 times/day<br>(1-2 days)	No decrease was shown with paromomycin + lactulose	of a controlled randomized trial. *Hepatogastroenterology*, **47,** 473-477 (2000).
7	CARBA-GLU® (carglumic acid)	100-250 mg/kg/day<br>2-3 times/day	The mean rate of changes in plasma ammonia from baseline to achievement of a normalised ammonia level (≤60 µM) was 272.8 µM/2.4 days<br>(~113.7 µM/day)<br>(from 36.5 to 352.9 µM/day)	Valayannopoulos, V., et al. Carglumic acid enhances rapid ammonia detoxification in classical organic acidurias with a favourable risk-benefit profile: a retrospective observational study. *Orphanet J. Rare Dis*. **11:**32 (2016 March 31). doi:10.1186/s13023-016-0406-2.
8	N-carbamyl-glutamate	100 mg/kg/day, in four divided doses<br>(3 days)	The mean decreasing in ammonia level was from 59 µM to 43 µM (over 3 days)<br>(~ 5.3 µM/day)	Ah Mew, N., et al. N-carbamylglutamate augments ureagenesis and reduces ammonia and glutamine in propionic acidemia. Pediatrics. 126, e208–e214 (2010). doi:10.1542/peds.2010-0008.
9	N-carbamyl-glutamate	100 mg/kg/day, in four divided doses<br>(3 days)	30 µM/3 days	Ah Mew, N., et al. Augmenting ureagenesis in patients with partial carbamyl phosphate synthetase 1<br>deficiency with N-carbamyl-L-glutamate. *J Pediatr*. **165,** 401-403 (2014).
10	Sodium benzoate	250 mg/kg over 2 h/day<br>Mean duration of treatment – 2 days (in all 10 days in the hospital).	Initial ammonia level in plasma - 245.5 (20.0–2274. 0) µM.<br>Final ammonia level at the end of treatment - 40 (13.0–181.0) µM.<br>Decrease - 102.75 µM/day.	Husson, M. C., et al. Efficacy and safety of i.v. sodium benzoate in urea cycle disorders: a multicentre retrospective study. *Orphanet J Rare Dis.* **11**:127 6 pages (2016). doi: 10.1186/s13023-016-0513-0.
11	Sodium benzoate	5 g<br>2 times/day<br>(11.6±6.4 days)<br><br>or lactulose 30 ml<br>3 times/day<br>(12.8±-9.1 days)	An arterial ammonia decrease in sodium benzoate group was from 64.6±34.2 µg/dl to 28.4±2 12.6 µg/dl,<br>in lactulose group – from 72.4±41.6 µg/dl to 18.6±24.2 µg/dl.<br>Thus, the rate of the ammonium decrease was about 3.1 and 5.7 µg/dl per day for sodium benzoate and lactulose, respectively (or 1.8 and 3.3 µM/day)	Sushma, S., et al. Sodium benzoate in the treatment of acute hepatic encephalopathy: a double-blind randomized trial. *Hepatology*, **16,** 138-44 (1992).

12	Sodiun phenylace-tate	9-12 g/day	Decrease in ammonium was 88±20 μM/7 days	Honda, *S.*, et al. Successful treatment of severe hyperammonemia using sodium phenylacetate powder prepared in hospital pharmacy. *Biol. Pharm. Bull.* **25,** 1244—1246 (2002).
13	Ammonul (solution of sodium phenylace-tate (10%) + sodium benzoate (10%))	250 mg/kg of sodium benzoate and 250 mg/kg of sodium phenylaceta te over 24 h	Decrease in ammonium was 100 μM/4 h	Ammonul. Treatment of acute hyperammonemia in patients with urea cycle disorders. Medical Review NDA 20-645 Center for Drug evaluation and Research, 2005.
14	L-ornithine-L-aspartate (Hepa Merz)	30 g daily in 500 mL of glucose 5% (over 4 h) for 7 days	Decrease in fasting and post-prandial ammonia up to 20 μM/4-7 days	Bai, M., et al. Randomised clinical trial: L-ornithine-L-aspartate reduces significantly the increase of venous ammonia concentration after TIPSS. *Aliment. Pharmacol. Therap*. **40,** 63-71 (2014).
15	Arginine<br><br>and citrulline	L-arginine (0.6 mmol/kg) or citrulline (1mmol/kg) 1 administ-ration	These preparations prevent an increase in ammonia during 4 h after alanine load (6.6 mM/kg) (without drugs administration there is an increase in ammonia up to 600 μM over 4 h)<br>Decrease of 200-350 μM/h	Mizutani, N., et al. Oral administration of arginine and citrulline in the treatment of lysinuric protein intolerance. *Tohoku J. Exp. Med*. **142,** 15-24 (1984).
16	L-carnitine	2 g<br>2 times/day (90 days)	In MHE, ammonia serum levels were decreased from 68.2±38.2 μM to 13.10 μM, 19.10 μM, and to 28.1 μM after 30, 60 and 90 days of treatment, respectively.<br>In HE1 – from 82.1±29.1 μM to 12.0 μM, 23.90 μM and to 41.00 μM after 30, 60 and 90 days of treatment, respectively.<br>In HE2, ammonia serum levels were decreased from 89.2±34.2 μM to 15.10 μM and to 36.00 μM after 60 and 90 days of treatment.<br>Thus, in all the decrease was 2.34-0.45 μM/day	Malaguarnera, M., et al. Effects of L-carnitine in patients with hepatic encephalopathy, *World J Gastroenterol*. **11,** 7197-7202 (2005).

References

1. Martinov, M. V., Plotnikov, A. G., Vitvitsky, V. M. & Ataullakhanov, F. I. Deficiencies of glycolytic enzymes as a possible cause of hemolytic anemia. *Biochim. Biophys. Acta* **1474,** 75-87 (2000). Available at: http://sci-hub.tw/https://doi.org/10.1016/S0304-4165(99)00218-4. Accessed May 12, 2018.
2. Manchester, K. L. Glutamate dehydrogenase: A reappraisal. *Biochem. Educ*. **13,** 131-133 (1985). Available at: http://sci-hub.tw/10.1016/0307-4412(85)90188-8. Accessed May 12, 2018.
3. Rife, J. E., & Cleland, W. W. Kinetic mechanism of glutamate dehydrogenase. *Biochemistry* **19,** 2321–2328 (1980). DOI: 10.1021/bi00552a007.
4. LeJohn, H. B., Suzuki, I. & Wright, J. A. Glutamate dehydrogenases of *Thiobacillus novellas*. Kinetic properties and a possible control mechanism. *J. Biol. Chem*. **243,** 118-128 (1968). Available at http://www.jbc.org/content/243/1/118.short. Accessed May 12, 2018.
5. Williamson, D. H., Lund, P. & Krebs, H. A. The redox state of free nicotinamide-adenine dinucleotide in the cytoplasm and mitochondria of rat liver. *Biochem. J.* **103,** 514-527 (1967). Available at: http://sci-hub.tw/https://doi.org/10.1042/bj1030514. Accessed May 12, 2018.
6. Bulos, B. & Handler, P. Kinetics of beef heart glutamic-alanine transaminase. *J. Biol. Chem*. **240,** 3283–3294 (1965). Available at: http://www.jbc.org/content/240/8/3283.full.pdf. Accessed May 12, 2018.
7. Grimshaw, C. E. & Cleland, W. W. Kinetic mechanism of *Bacillus subtilis* L-alanine dehydrogenase. *Biochemistry* **20,** 5650–5655 (1981). DOI: 10.1021/bi00523a002.
8. Meek, T. D. & Villafranca, J. J. Kinetic mechanism of *Escherichia coli* glutamine synthetase. *Biochemistry* **19,** 5513–5519 (1980). DOI**:** 10.1021/bi00565a008.
9. T. D. Meek, T. D., Johnson, K. A. & Villafranca, J. J. *Escherichia coli* glutamine synthetase. Determination of rate-limiting steps by rapid-quench and isotope partitioning experiments. *Biochemistry* **21,** 2158–2167 (1982). DOI: 10.1021/bi00538a027.
10. Sanz, S., Lizano, C., Luque, J. & Pinilla, M. In vitro and in vivo study of glutamate dehydrogenase encapsulated into mouse erythrocytes by a hypotonic dialysis procedure. *Life Sci*. **65,** 2781-2789 (1999). Available at: http://sci-hub.tw/https://doi.org/10.1016/S0024-3205(99)00546-9. Accessed May 12, 2018.
11. Kruckeberg, W. C., Sander, B. J., Sillivan, D. C. Plasmodium berghei: glycolytic enzymes of the Infected mouse erythrocyte. *Exp. Parasitol*. **51,** 438-443 (1981).

12. Morimoto, M., Kanno, H., Asai, H., Tsujimura, T., Fujii, H., Moriyama, Y., Kasugai, T., Hirono, A., Yuzou Ohba, Y., Miwa, S., Kitamura Y. Pyruvate kinase deficiency of mice associated with nonspherocytic hemolytic anemia and cure of the anemia by marrow transplantation without host irradiation. *Blood* **86,** 4323-4330 (1995).
13. Magnani, M., Rossi, L., Stocchi, V., Cucchiarini, L., Piacentini, G., Fornaini, G. Effect of age on some properties of mice erythrocytes. *Mech. Ageing Dev*. **42,** 37-47 (1988).
14. Rivera, S., Lopez-Soriano, F. J., Azcon-Bieto, J., Argiles, J. M. Blood amino acid compartmentation in mice bearing Lewis lung carcinoma. *Cancer Res*. **47,** 5644-5646 (1987).
